# Fast and Ultra-Fast mRNA-Engineered Anti-CA125/MUC16 CAR-T-Cells: An In Vitro Study

**DOI:** 10.3390/biomedicines14102147

**Published:** 2026-09-23

**Authors:** Kamilla Aminulla, Artem Pilunov, Alexandra Maleeva, Tatiana Kulinich, Vladimir Bozhenko

**Affiliations:** Russian Scientific Center of Roentgen Radiology, 117997 Moscow, Russia; kamilla.ami@mail.ru (K.A.); maleevainform@gmail.com (A.M.); sobral@mail.ru (T.K.); vbojenko@mail.ru (V.B.)

**Keywords:** CAR T, immunotherapy, solid tumor, ovarian cancer, mRNA

## Abstract

**Background**: Ovarian cancer treatment is frequently complicated by postoperative recurrence associated with metastatic dissemination and acquired chemotherapy resistance, highlighting the need for new therapeutic approaches. Messenger RNA (mRNA)-based chimeric antigen receptor (CAR) T-cell therapy is a promising strategy for solid tumors because it enables transient CAR expression and may shorten the manufacturing process compared with lentiviral approaches. Given the potential relevance of rapid, transient CAR expression to locoregional or adjuvant applications, we evaluated an mRNA-based CAR-T cell approach targeting cancer antigen 125 (CA125) in vitro. **Methods**: CAR-T cells were generated from either activated or nonactivated (“resting”) lymphocytes obtained from healthy donors using mRNA electroporation. Their activity against target tumor cells was assessed by impedance-based real-time cell analysis (RTCA) using the xCELLigence platform. **Results**: We compared two mRNA CAR-T cell manufacturing strategies: “fast” CAR-T cells generated using a 3-day activation-based workflow and “ultra-fast” CAR-T cells generated from nonactivated T cells and functionally evaluated within 24 h after electroporation. “Fast” CAR-T cells exhibited strong in vitro activity, including at an effector-to-target ratio of 1:4, representing a high target-cell burden. “Ultra-fast” CAR-T cells were also functional but showed lower activity, with an observable effect requiring an effector-to-target ratio of at least 2.5:1. **Conclusions**: These findings support the feasibility of rapid mRNA-engineered anti-CA125 CAR-T cell manufacturing using either activated or nonactivated T cells. The two approaches differ in functional activity and may therefore be suitable for different manufacturing workflows and intended applications.

## 1. Introduction

Ovarian cancer ranks among the most prevalent malignancies and is a leading cause of cancer-related mortality in women [1]. Standard treatment involves surgical intervention combined with chemotherapy [2], which achieves remission in most patients [3]. However, recurrence is frequent, especially in advanced stages, with rates varying by disease stage and treatment context [4,5]. Effective therapeutic options for recurrent disease remain limited because tumors frequently acquire chemotherapy resistance, while disseminated disease may preclude further surgery [4,6]. Consequently, developing effective strategies to prevent and manage ovarian cancer recurrence is a critical priority. Chimeric antigen receptor T-cells (CAR-T-cells) are a promising therapeutic strategy for ovarian cancer [7]. Although CAR-T therapy was first shown to be highly effective in leukemia, its use in solid tumors, including ovarian cancer, is expanding [8]. Multiple immunotherapy targets have been identified for ovarian cancer, including mesothelin [9], folate receptor-α [10], human epidermal growth factor receptor-2 (HER2) [11], and mucin glycoprotein 16 (MUC16) [9,10,11,12]. Among these, MUC16 is one of the most extensively studied targets for CAR-T cell development [12,13], as its expression correlates with disease progression and increases substantially at stage 4, when immunotherapy becomes particularly relevant [14]. The transmembrane form of MUC16 contains a large extracellular domain with multiple tandem repeats and CA125-reactive epitopes, providing a tumor-associated surface target for CAR recognition [12,15]. However, MUC16 expression is not restricted to malignant tissues [16,17], and the extent to which its polarized distribution limits CAR-T-cell access in healthy tissues remains a hypothesis rather than an established safety feature. Consequently, tumor expression alone cannot establish the safety of MUC16-directed CAR-T therapy. Although CA125-reactive epitopes have been mapped within the extracellular repeat region of MUC16, the exact epitope recognized by the CAR used in this study remains unknown [12,15]. MUC16 can undergo proteolytic cleavage, releasing soluble CA125 antigen, which is detectable in the blood and serves as a key diagnostic marker for ovarian cancer [18,19]. Because soluble CA125 may compete with cell-surface MUC16 for CAR binding, future research should investigate the impact of clinically relevant concentrations of serum- or ascites-derived CA125 on CAR-T cell activity [20]. Under normal physiological conditions, MUC16 serves a protective function in mucosal tissues. In cancer, MUC16 is frequently overexpressed and plays a significant role in tumor cell biology. MUC16 facilitates mesothelin-mediated cell adhesion [21]. Downregulation of MUC16 on the surface of OVCAR-3 cancer cells promotes epithelial-mesenchymal transition [22]. Conversely, high MUC16 expression may benefit cancer cells by inhibiting immune synapse formation, thereby contributing to immune evasion [23]. Although preclinical results are encouraging, CAR-T therapy for solid tumors faces significant technical challenges, such as the immunosuppressive tumor microenvironment, antigen heterogeneity, and limited tumor infiltration by CAR-T-cells [24]. Recent strategies to overcome these obstacles include incorporating chemokine receptors into CAR-T cells to enhance tumor-specific migration [25] and suppressing checkpoint molecules in CAR-T cells [26]. Many experimental and clinical CAR-T products still employ conventional CAR architectures, while additional modifications are under investigation to address barriers specific to solid tumors [24,27,28]. Optimizing delivery methods may be crucial for improving CAR-T therapy efficacy [29]. Because ovarian cancer commonly disseminates within the peritoneal cavity, intraperitoneal administration is being investigated to increase local effector-cell exposure to peritoneal disease and residual tumor deposits [30]. In a preclinical model of peritoneal carcinomatosis, intraperitoneal CAR-T-cell delivery was more effective than intravenous administration [31]. These observations provide a rationale for exploring CAR-T therapy after cytoreductive surgery, when the remaining disease burden may consist predominantly of residual peritoneal deposits; however, this postoperative application remains hypothetical and was not modeled in the present in vitro study. mRNA electroporation offers an attractive nonviral approach by enabling transient CAR expression and potentially shortening manufacturing time compared to some viral workflows [32,33]. Permanently expressed CARs in T cells may increase the risk of off-tumor toxicity [34,35], as many tumor-associated antigens are also present in healthy tissues. By limiting the duration of CAR expression, mRNA modification may limit the duration of target recognition; however, this is a theoretical safety consideration rather than a safety advantage demonstrated in the present study. Acute cytokine-mediated or on-target, off-tumor effects may still occur while the CAR is expressed. Currently, mRNA transfection-based CAR-T production is less common than viral transduction or plasmid DNA transfection [27]. Production methods for mRNA-CAR-T cells vary widely across published protocols, whereas dominant lentiviral CAR-T technologies (such as Kymriah and Yescarta) follow a more standardized approach. Therefore, optimizing mRNA-CAR-T production is essential for advancing cancer immunotherapy. Using nonactivated T cells addresses a distinct manufacturing trade-off: omitting activation and expansion can reduce production time to about one day [36], whereas activation can substantially alter T-cell phenotype and function [37]. A direct comparison is therefore needed to determine whether manufacturing-time savings are accompanied by changes in immediate functional activity. Our previous work evaluated the antitumor activity of anti-CA125 CAR-T cells generated using an earlier platform, while the present study focuses on mRNA-based transient modification, rapid manufacturing, and comparison of activated and resting T cells [38].

## 2. Materials and Methods

### 2.1. T-Cell Separation and Activation

The study was conducted in accordance with the Declaration of Helsinki and received approval from the Institutional Ethics Committee of the Russian Scientific Center of Roentgen Radiology of the Ministry of Health of the Russian Federation, meeting No. 4 held on 28 April 2023. The Ethics Committee authorized blood donation for CAR-T manufacturing for the duration of the state assignment. Peripheral blood mononuclear cells (PBMCs) were collected from healthy donors following informed consent. Inclusion criteria specified that donor serum must be negative for HIV and HBV antibodies. Samples were obtained from 20 donors (aged 25–50 years, both male and female). Donor relevance to specific assays is detailed in Supplementary Table S1. Blood samples were collected from January to September 2025 in bags (JMS Singapore PTE LTD, Singapore) containing 25–40 mL of blood concentrate with anticoagulant. The buffy coat was isolated by centrifugation at 750 g for 20 min using a Ficoll density gradient (PanEco, Moscow, Russia). The resulting pellet was washed three times with phosphate-buffered saline (PBS), pH 7.4 (PanEco) at 350 g for 10 min, 160 g for 15 min, and 300 g for 10 min, respectively. The number and composition of isolated cells were assessed using a Sysmex XN-550 hematology analyzer Ver. 5 (Sysmex, Kobe, Japan). Isolated PBMCs were aliquoted at 50 × 10^6^ cells per vial and cryopreserved in liquid nitrogen in RPMI-1640 medium (PanEco) supplemented with 10% fetal bovine serum (Globe Kang, Qinhuangdao, China), 10 μg/mL gentamicin (PanEco), and 10% DMSO (PanEco). Prior to in vitro studies, cells were thawed in a 37 °C water bath and cultured in complete RPMI-1640 growth medium with 10% FBS and 10 μg/mL gentamicin at 37 °C and 5% CO_2_. For experiments comparing cryopreserved and fresh cells, paired samples from the same donors were prepared: some samples underwent brief cryopreservation (2 h in liquid nitrogen), while others were processed without cryopreservation and cultured overnight in parallel with the thawed cells. CD3+ cells were isolated from thawed PBMC aliquots using magnetic columns and the Human CD3+ Cell Separation Kit (RWD Life Science Co., Ltd., Shenzhen, China) according to the manufacturer’s instructions. Following thawing, cells were incubated overnight to facilitate recovery. Pre-filtered and concentrated PBMCs were diluted with PBS containing 0.5% bovine serum albumin (BSA) (PanEco) and 2 mM EDTA (PanEco) to a concentration of 1 × 10^8^ cells/mL, then incubated with biotinylated anti-CD3 antibodies for 10 min at 4 °C. After washing and centrifugation, cells were incubated with streptavidin-conjugated magnetic beads for 15 min at 4 °C. The cells were then applied to a pre-rinsed magnetic column for magnetic separation. The purity of the CD3+ fraction was at least 97% (Supplementary Figure S1). Activation of PBMCs or separated CD3+ cells was performed immediately after separation for CD3+ cells, or 16 h after thawing for PBMCs, by adding Dynabeads Human T-Expander CD3/CD28 magnetic particles (Thermo Fisher Scientific Inc., Waltham, MA, USA) at a 1:1 ratio to the total cell count in complete RPMI-1640 medium supplemented with IL2 (Biotech LLC, St-Petersburg, Russia) at a concentration of 200 U/mL. Cells were seeded in T25 flasks at a starting density of 5 × 10^6^ cells/mL. Transfection was conducted on the second day after activation. Magnetic beads were removed the day after electroporation using a magnet.

The study was conducted in accordance with the Declaration of Helsinki and approved by the Institutional Ethics Committee of the Russian Scientific Center of Roentgen Radiology of the Ministry of Health of the Russian Federation, meeting No. 4 held on 28 April 2023. The Ethics Committee approved blood donation for CAR-T manufacturing for the duration of the state assignment. Peripheral blood mononuclear cells (PBMCs) were obtained from healthy donors after informed consent. Inclusion criteria required serum to be negative for HIV and HBV antibodies. The study used samples from 20 donors aged 25–50 years, both male and female. The relevance of each donor to a specific assay could be accessed in Supplementary Table S1. The blood samples were collected from January to September 2025 in bags (JMS Singapore PTE LTD, Singapore) containing 25–40 mL of blood concentrate with an anticoagulant. The buffy coat was isolated by centrifugation at 750 *g* for 20 min in a Ficoll density gradient (PanEco, Russia). Then, the pellet was washed three times with phosphate-buffered saline (PBS), pH 7.4 (PanEco, Russia) at 350 g for 10 min, 160 g for 15 min, and 300 g for 10 min, respectively. The number and composition of isolated cells were monitored on a Sysmex XN-550 hematology analyzer (Sysmex, Japan). After isolation, PBMCs were aliquoted as 50 × 10^6^ cells per vial and cryopreserved in liquid nitrogen in RPMI-1640 medium (PanEco, Russia) containing 10% fetal bovine serum (Globe Kang, China), 10 μg/mL gentamicin (PanEco, Russia), and 10% DMSO (PanEco, Russia). Before in vitro studies, cells were thawed in a 37 °C water bath and cultured in complete RPMI-1640 growth medium with 10% FBS and 10 μg/mL gentamicin at 37 °C and 5% CO_2_. For the experiment comparing cryopreserved and fresh cells, paired samples from the same donors were created: some samples underwent brief cryopreservation (2 h in liquid nitrogen), and the others were processed further without cryopreservation and cultured overnight in parallel with the thawed cells.

CD3+ cells were separated from thawed PBMC aliquots using magnetic columns with the Human CD3+ Cell Separation Kit (RWD Life Science Co., Ltd., China) according to the manufacturer’s instructions, with cells incubated at least overnight after thawing to allow recovery. Pre-filtered and concentrated PBMCs were diluted with PBS containing 0.5% bovine serum albumin (BSA) (PanEco, Russia) and 2 mM EDTA (PanEco, Russia) to 1 × 10^8^ cells/mL and incubated with biotinylated anti-CD3 antibodies for 10 min at 4 °C. After washing with buffer and centrifugation, the cells were incubated with streptavidin-conjugated magnetic beads for 15 min at 4 °C. After washing, the cells were applied to a pre-rinsed magnetic column, and magnetic separation was performed. The CD3+ fraction purity was at least 97% (Supplementary Figure S1).

Activation of PBMC or separated CD3+ cells was performed immediately after separation in the case of CD3+ cells or 16 h after thawing for PBMC by adding Dynabeads Human T-Expander CD3/CD28 magnetic particles (Thermo Fisher Scientific Inc., USA) at a 1:1 ratio to the total cell count in complete RPMI-1640 medium supplemented with IL2 (Biotech LLC, Russia) at a concentration of 200 U/mL. Cells were seeded in a T25 flask at a starting density of 5 × 10^6^ cells/mL. Transfection was performed on the 2nd day after activation. We removed the beads the day after electroporation using a magnet.

### 2.2. Transfection

For lymphocyte transfection, mRNA encoding green fluorescent protein (GFP), the 3CA2-CAR3g plasmid, or mRNA encoding the third-generation CAR specific for CA125 [38] was used. The CAR sequence included defined scFvs from the immunoglobulin kappa light chain (IgK) and immunoglobulin heavy chain (IgH) connected by a hinge, a transmembrane domain from CD8, and signaling domains from CD28, 4-1BB, and CD3ζ. The N-terminus contained an altered leader peptide sequenceµ MDMRVPAQLLGLLLLWLRGARC from the IgK V-III region [39], which was introduced into the previous CAR construct to increase surface expression of CAR. The plasmid and mRNA contained identical CAR sequences. The CAR scheme is presented in Supplementary Figure S2. All mRNAs were provided by the Gamaleya National Research Center for Epidemiology and Microbiology and were synthesized in vitro. All mRNAs contained N1-methyl-pseudouridine instead of uridine, were 5′-capped with CleanCap AG, and 3′-polyadenylated.Lymphocytes were transfected by electroporation using the 4D-Nucleofector System (Lonza, Basel, Switzerland) with the X Unit module, applying pulse code FI-115 for plasmid transfection of resting cells and EO-115 for all other cases. Previous research demonstrated that FI-115 is more efficient for plasmid modification of resting cells, although it results in lower viability [39]. Therefore, plasmid transfection results may be partially confounded by differences in pulse code, with FI-115 being more effective. Supplementary Figure S3 presents the CAR-T manufacturing scheme from activated and resting T-cells. For each sample, 100-μL cuvettes with the V4XP-3024 P3 Primary Cell 4D-Nucleofector Kit L were used, following the manufacturer’s protocol. For each sample, 5 × 10^6^ cells were resuspended in 100 μL of electroporation buffer, and 5 μg of plasmid or 5–20 μg of mRNA were added. The mRNA concentration was 2 μg/μL, and the plasmid concentration was 1 μg/μL; the total volume of transfection medium did not exceed 110 μL. For mRNA transfection, Ribolock RNase inhibitor (Thermo Fisher Scientific) was used at a final concentration of 1 unit/μL in the electroporation buffer; no RNase inhibitor was added to the post-transfection culture medium. As a negative control, cells were electroporated without plasmid or mRNA (pulse control). After transfection, cells were incubated in a 6-well plate in complete growth medium (for activated cells, 200 U/mL of IL2 was added) at 37 °C and 5% CO_2_ for 20–24 h.

### 2.3. Flow Cytometry Analysis

Expression of GFP and anti-CA125 CAR was assessed using flow cytometry. Expression of CA125-specific CAR on lymphocytes was determined by two-step staining. To a sample of 0.5 × 10^6^ cells resuspended in 100 μL of staining buffer (PBS, 0.5% BSA, 2 mM EDTA), 1 μL of biotinylated CA125 antigen (concentration 1.33 μg/μL, R222b lot 2311, XEMA, Moscow, Russia) was added and incubated for 20 min. After washing twice with staining buffer, 50 μL of diluted PE-conjugated streptavidin (cat. No. 2626015, 0.056 μg/mL, Sony Biotechnology, Tokyo, Japan). Analysis was performed on a FongCyte flow cytometer (Challenbio, Beijing, China). Supplementary Figure S4 shows the gating strategy. Sequential gating on lymphocyte populations (forward and side scatter) and exclusion of dead cells (DAPI-negative) enabled us to estimate the proportion of GFP^+^ or CAR-CA125 cells among living lymphocytes. For the T cell phenotype analysis, the following antibodies were used: CD3-FITC/CD4-PE dual-color reagent (cat. No. A07733); CD3-FITC/CD8-PE dual-color reagent (cat. No. A07734), CD25-PE (Cat. No. IM0479U); CD69-PE (Cat. No. IM1943U) (Beckman-Coulter, Brea, CA, USA), and PD1-PerCP Cy5.5 (Cat. No. 2249570, Sony Biotechnology, Japan).

### 2.4. Cell Lines

To evaluate the in vitro efficacy and specificity of the resulting CAR-T cells, human CA125-positive OVCAR-3 and surface MUC16-negative OAW-42 ovarian adenocarcinoma cell lines were used. OVCAR-3 (cryovial 300307) and OAW-42 (cryovial 300304) were purchased from Cell Lines Service GmbH in November 2016. The expression level of MUC16 was measured using flow cytometry (Supplementary Figure S5). The initial aliquot was cryopreserved at passage 4 for OVCAR-3 and passage 5 for OAW-42. After thawing, cells were passaged 2-4 times before cytotoxic assays. The identity of the cell lines was confirmed by STR analysis using the ABI 3500 Genetic Analyzer (Applied Biosystems, Waltham, MA, USA). The absence of mycoplasma contamination was monitored using the Mycoreport PCR kit (Evrogen, Moscow, Russia). OVCAR-3 cells were cultured in RPMI-1640 medium supplemented with 20% FBS, 10 μg/mL gentamicin, and 0.3 U/mL insulin (Medsintez, Ekaterinburg, Russia), and OAW-42 cells were cultured in DMEM (glucose content 4.5 g/L; PanEco) supplemented with 10% FBS, 10 μg/mL gentamicin, and 0.02 U/mL insulin.

### 2.5. Real-Time Proliferation Assessment

The XCELLigence real-time cell analysis (RTCA) technology (ACEA Biosciences, San Diego, CA, USA) monitors the population dynamics of viable adherent cells in real time by measuring changes in the “cell index,” which directly correlates with the number of cells attached to a surface. Each RTCA experimental point was performed in two technical replicates. For cytotoxicity analysis, target cells were cultured in E-plate 16 plates (Agilent, Santa Clara, CA, USA) for 24 h to reach 30–50% confluence (~10–15,000 cells were seeded per well in 100 μL of complete growth medium). Effector cells (CAR-T cells or pulsed lymphocytes) were then added to the wells in 100 μL of complete growth medium RPMI1640 for OVCAR-3 or DMEM for OAW-42. Although DMEM is not considered an optimal medium for long-term T cell culture, T cells can exert cytotoxicity in DMEM without significant handicap [40,41]. The effector:target (E:T) ratio varied from 0.25:1 to 5:1 (2500 to 50,000 cells, respectively). The E:T ratios were calculated based on the total input of effector cells. Controls included wells seeded with cancer cell lines alone; OVCAR-3 cultured with CAR-T; OVCAR-3 incubated with T cells expressing an irrelevant CAR (specific for carcinoembryonic antigen (CEA)); or T cells alone. Additionally, we performed an antigen-blocking experiment. To the wells with OVCAR, soluble CA125 antigen was added (concentration 1.33 μg/μL, R222b lot 2311, XEMA) to a final concentration of 25 μg/mL. This is the same antigen as is used for CAR staining. Before co-culturing with OVCAR, T cells were cultured with soluble antigen at 25 μg/mL for 30 min and washed once. The final medium volume in all wells was 200 μL.

### 2.6. CCK-8 Assay

For the CCK-8 assay, target cells were cultured in a 96-well plate for 24 h to reach 30–50% confluence (~10–15,000 cells/well). Effector cells (CAR-T lymphocytes or pulsed lymphocytes) were then added to the wells in 100 μL of complete growth medium, RPMI1640 for OVCAR-3 or DMEM for OAW-42, to obtain an effector:target (E:T) ratio of 1:1 for activated cells and 5:1 for resting cells. Tumor cell lines served as controls; 100 μL of culture medium was added to each well. All experimental conditions were tested in technical duplicate. After 24 h, 100 μL of supernatant was collected for ELISA. Then, 10% (*v*/*v*) of Cell Counting Kit-8 (CCK-8) reagent (Thermo Fisher Scientific) was added to the remaining medium. After a 3 h incubation, absorbance was measured at 450 nm with a reference wavelength of 650 nm using a SpectraMax i3 reader (Molecular Devices, San Jose, CA, USA).

### 2.7. ELISA

The concentration of TNF-α in the supernatants collected in Section 2.6 was determined using the alpha-TNF-EIA-BEST kit (Vector-Best, Novosibirsk, Russia) according to the manufacturer’s instructions. Supernatants were added at 40 μL per well to a plate pre-coated with immobilized monoclonal anti-TNF-α antibodies in technical duplicates. The optical density was measured at a primary wavelength of 450 nm and a reference wavelength of 630 nm using a SpectraMax i3 reader (Molecular Devices). We calculated the concentration of TNF-α in the studied samples using a standard curve.

### 2.8. IFN-γ/IL-2/Granzyme B Three-Color FluoroSpot Assay

The frequencies of IFN-γ-, IL-2-, and granzyme B-secreting T cells were determined using a human IFN-γ/IL-2/Granzyme B Three-Color FluoroSpot assay (Cellular Technology Limited, Shaker Heights, OH, USA) according to the manufacturer’s protocol. Briefly, activated and resting T cells after electroporation without or with CAR-encoding mRNA were cultured with the OVCAR-3 target cell line or the OAW-42 control cell line, as described above, for 5 or 10 h. Before the assay, the cells were washed and resuspended in CTL Test Medium (Cellular Technology Limited) without exogenous IL-2.

Phytohemagglutinin, PHA (Paneco) at a concentration of 5 µg/mL was used as a positive control. All experimental conditions were tested in technical duplicate. At the 5 h time point, pulse-only and CAR mRNA-electroporated cells were evaluated, whereas at the 10 h time point only CAR mRNA-electroporated cells were analyzed. Following incubation, the plates were washed, and captured IFN-γ, IL-2, and granzyme B were detected using the corresponding detection antibodies and fluorochrome-conjugated reagents according to the manufacturer’s protocol.

After completion of the detection procedure, the plates were scanned using an ImmunoSpot S6 Ultra Analyzer (Cellular Technology Limited). Fluorescent spots corresponding to each analyte were enumerated using ImmunoSpot 7 software. Results were normalized to the number of effector T cells plated and expressed as spot-forming units (SFU) per 10^4^ effector T cells.

### 2.9. Statistical Analysis

We used GraphPad Prism 9 for the analysis. We assessed statistical significance using a Wilcoxon signed-rank test, as this method was most feasible under the investigated conditions: small sample size, paired samples, and non-normal distribution. We set the significance threshold at 0.05 (*p* < 0.05). For the RTCA assay, we calculated the area under the curve (AUC) for each technical replicate for the 60-h RTCA measurement. We then averaged two technical replicates for each donor and plotted AUC values independently for each donor. To assess statistical differences between RTCA curves, we used two-way ANOVA (mixed model) with the Geisser-Greenhouse correction to account for unequal variability in the differences.

GraphPad Prism 9 software was used for the analysis. Statistical significance was assessed using a Wilcoxon signed-rank test. We set the significance threshold at 0.05 (*p* < 0.05). For the RTCA assay, we calculated the area under the curve (AUC) for each technical replicate for the 60 h RTCA measurement. We then averaged two technical replicates for each donor and plotted AUC values independently for each donor. To measure statistical differences between RTCA curves, we used two-way ANOVA (mixed model) with the Geisser-Greenhouse correction.

## 3. Results

### 3.1. The Use of RNase Inhibitors Increases Transgene Expression

An essential requirement for efficient CAR-T therapy is achieving a high proportion of modified cells. Extracellular RNA is vulnerable to enzymatic degradation in biological fluids, and RNA-degrading activity can influence the stability of exogenous mRNA [42]. To evaluate the effect of an RNase inhibitor on mRNA transfection efficiency, cryopreserved peripheral mononuclear cell samples from six healthy donors were electroporated with GFP-encoding mRNA the day after thawing, either with or without an RNase inhibitor in the electroporation buffer. The inclusion of RNase inhibitors modestly increased the percentage of GFP-positive lymphocytes, from an average of 60% to 85% (Figure 1A). Notably, the median fluorescence intensity (MdFI) increased more than threefold, from approximately 1800 to 6500 units (Figure 1B), indicating enhanced integrity of mRNA delivered into cells. The presence of RNases in the electroporation medium significantly affects mRNA electroporation efficiency, supporting the use of an RNase inhibitor under these transfection conditions. Consequently, an RNase inhibitor was included in all subsequent experiments.

### 3.2. Impact of Cryopreservation on the mRNA Electroporation Efficiency

Cryopreserved leukapheresis material is commonly utilized for CAR-T cell production because it allows for convenient storage and enables more flexible administration of CAR-T therapy. The majority of studies indicate that the use of frozen cells for CAR-T cell production does not significantly impact the quality of the CAR-T product [43]. While survival and proliferative potential are slightly reduced [44], as is cytokine secretion [45], the efficacy and persistence of CAR-T cells in vivo derived from both fresh and frozen material are comparable [46]. Paired cryopreserved and fresh PBMCs from three healthy donors were transfected with mRNA encoding GFP or CAR. Transfection was performed the next day after thawing, as immediate electroporation negatively impacts cell viability [47]. The percentage of GFP-positive cells did not differ between fresh and cryopreserved samples and reached 90% (Figure 2A). At the same time, the median fluorescence was approximately one and a half times higher in cells not subjected to cryopreservation (Figure 2B). CAR-modified cryopreserved cells demonstrated greater variability between samples than GFP-modified samples. However, although the trends observed in our exploratory study align with other published data, the sample size of three donors is too small to reach statistical significance. Nevertheless, cryopreserved material remained compatible with the tested mRNA electroporation and expression protocol.

### 3.3. The Effect of T-Cell Activation on mRNA Modification Efficiency

T-cell activation is a standard procedure used in most CAR-T production protocols, including transfection of T-cells with CAR-encoding mRNA [34]. In vitro T-cell activation requires three signals (TCR, costimulatory molecules, and cytokine receptors) and fundamentally alters cellular processes, activating metabolism and stimulating proliferation [48]. We transfected activated and resting PBMCs from six healthy donors with mRNA encoding GFP or CAR. Activation significantly increased the transfection efficiency: compared to resting cells, the percentage of GFP+ cells increased to nearly 100%, and the average percentage of CAR+ cells increased from 75% to 90% (Figure 3A). The CD4/8 ratio in samples from the two donors studied remained stable after activation and transfection with CAR-encoding mRNA or plasmid, or pulse control (Supplementary Figure S6). Similarly, electroporation did not affect the CD4/CD8 ratio of resting T-cells. However, electroporation increased CD69 expression in resting cells, suggesting some stress-related activation. As expected, activation increased the levels of CD69-, CD25-, and PD-1-positive cells, and electroporation of activated cells did not affect these protein levels. Furthermore, the median fluorescence intensity for lymphocytes transfected with GFP mRNA was increased more than fivefold upon activation (Figure 3B). At the same time, activation had no apparent effect on CAR expression levels. The observed discrepancy between GFP and CAR likely reflects the more complex processing required for CAR at the cell surface, whereas GFP does not require surface presentation to contribute to the detected signal. Due to its larger size and complexity, CAR must traverse the endoplasmic reticulum and Golgi apparatus for accurate folding, glycosylation, and transport, potentially limiting its surface expression. Additionally, increased tonic signaling and CAR internalization in activated lymphocytes may counteract the effect of activation on CAR surface levels. The precise mechanisms underlying this phenomenon remain undetermined and are not addressed in the current research.

We monitored CAR expression dynamics in activated and resting PBMCs from one donor for one week after transfection with CAR-encoding mRNA and plasmid (Supplementary Figure S7). The most rapid transgene decline was observed in mRNA-modified activated T-cells—CAR was practically undetectable on day 4; plasmid-modified activated cells retain detectable expression a day longer. The degradation of the transgene in resting T-cells takes longer—mRNA-derived and plasmid-derived CAR expression can still be detected on the 5th and 6th days post-transfection, respectively. The faster decline of transgene expression in activated cells can be explained by their rapid proliferation, while the transgenic vector cannot replicate; its concentration dilutes with every cell division. T-cell activation is a necessary step in CAR-T cell production and is used in most products, including “fast” CAR-T protocols that use non-viral delivery methods [43]. Rapid manufacture of resting CAR-T cells has been reported using other gene-delivery systems [36,37], while mRNA-based CAR-T protocols have also been described [32,34,49]. To our knowledge, the combination of resting T-cells, mRNA electroporation, and the present anti-CA125 CAR has not been systematically evaluated.

### 3.4. Activated mRNA CAR-T Cells Demonstrate Better Cytotoxicity Compared to Resting CAR-T Cells

Activated and resting CD3+ lymphocytes from a healthy donor were transfected with mRNA encoding a CAR specific for the CA125 antigen at a dose of 20 μg per transfection. The day after transfection, lymphocytes were added to OVCAR ovarian cancer cells expressing high levels of CA125, as well as to surface MUC16-negative OAW-42 ovarian cancer cells (Figure 4). Lymphocytes were co-cultured with tumor cells at effector:target (E:T) ratios of 5:1, 2.5:1, and 1:1, and changes in cancer cell viability were measured by impedance using an Agilent XCELLigence system. Resting CAR-T cells exhibited cytotoxicity towards OVCAR-3 cells at E:T ratios of 5:1 and 2.5:1 (Figure 4A). Activated CAR-T cells demonstrated a significantly more pronounced cytotoxic effect, with complete loss of adherent tumor-cell signal for OVCAR-3 cells observed even at the lowest E:T ratio of 1:1. Under these conditions, CAR-T cells were active against OVCAR-3 but not against OAW-42 (Figure 4B), which lacks surface CA125/MUC16 expression.

### 3.5. mRNA-CAR-T, but Not Plasmid CAR-T Cells, Exert Cytotoxicity Under High Target-Cell Burden

Plasmid DNA transfection is widely used in gene therapy and for generating CAR-T cells; however, its efficacy is limited by the high toxicity of plasmids [33]. Viability, transfection efficiency, and MdFI were compared between plasmid and mRNA vectors encoding a CA125-specific CAR across a concentration range of 1–20 μg per transfection for activated and resting T-cells derived from one donor (Supplementary Figure S8). Increasing the dose increased MdFI for both mRNA and plasmid. However, the dynamics of modification efficiency and viability differed substantially between mRNA and plasmid. For mRNA, modification efficiency increased continuously, and viability remained stable in both activated and resting cells. In contrast, plasmid modification efficiency peaked at 5 μg per transfection and declined at higher doses, with viability decreasing steadily as plasmid dose increased. CAR-T cells were added to OVCAR cells at a 5:1 ratio for resting lymphocytes and a 1:1 ratio for activated lymphocytes (Figure 5A). The number of CAR-T cells added was normalized to the post-transfection viability percentage, and vector amounts were selected to maximize transgene expression while maintaining acceptable viability (5 μg plasmid and 20 μg mRNA per transfection). Different quantities were intentionally used; although this approach does not allow a direct comparison of the cytotoxic efficiency of mRNA- and plasmid-generated CAR-T cells, it enables estimation of their efficiency under feasible manufacturing conditions. Based on the exploratory study in Figure 4, the E:T ratio was fixed at 5:1 for resting cells and 1:1 for activated cells, as these ratios yield a comparable rate of signal reduction for RTCA. The following results are therefore restricted to within-group analysis. In the initial exploratory experiment, CAR-T cells generated from resting cells using plasmids failed to suppress tumor cell growth at a 5:1 E:T ratio, whereas mRNA-CAR-T cells were efficient under these conditions. Activated cells exhibited cytotoxicity regardless of the gene-delivery method; however, mRNA-CAR-T cells demonstrated a much faster cytotoxic effect, as indicated by the curve shape. The faster activity observed with mRNA may reflect differences in CAR expression, viable-cell recovery, or cellular responses to the two nucleic-acid formats; these mechanisms were not directly assessed in this study.

The cytotoxicity of CA125-specific CAR-T cells derived from the same donor as in the previous experiment was evaluated under high target cell burden conditions at E:T ratios below 1. Activated mRNA-modified CAR-T cells demonstrated superior cytotoxicity compared to plasmid-modified CAR-T cells (Figure 5B). These findings indicate that mRNA-modified activated CAR-T cells exhibit high cytotoxicity against ovarian cancer cells expressing high levels of CA125 and retain efficiency under high target cell burden.

Following the establishment of an effective range for CAR-T cells in exploratory experiments, CAR-T products were manufactured for three additional donors to validate the specificity of cytotoxic activity. Resting and activated CD3+ cells were transfected with 20 μg of mRNA or 5 μg of plasmid DNA. Mock-electroporated cells (pulse-control), cells modified with a plasmid encoding an irrelevant CAR specific for CEA, and an antigen-blocking control with soluble CA125 antigen (added to the culture medium as described in Section 2.5) were included. Figure 6 presents the averaged curves across three donors for mRNA-CAR-T cells and two donors for plasmid-derived CAR-T cells. Supplementary Figure S9 shows curves for individual donors, and Supplementary Figure S10 shows calculated AUCs. Activated and mRNA-modified resting cells demonstrated stable, efficient functional antitumor activity against OVCAR-3 cells. Activated T-cells are known to acquire the phenotype of lymphokine-activated killers [50], contributing to CAR-independent cytotoxicity in this assay. Although activated T-cells exhibited some CAR-independent antitumor activity, the differences between the CAR-dependent and CAR-independent RTCA curves (activated pulse control and irrelevant CAR) were statistically significant (2-way ANOVA, row factor × column factor; row factor; subject—*p* < 0.0001; column factor *p* = 0.01). Resting T-cells showed significantly less CAR-independent cytotoxicity and a more pronounced difference between mRNA-CAR cells and pulse control (2-way ANOVA, row factor × column factor; row factor; subject—*p* < 0.0001; column factor *p* = 0.0042). No cytotoxic effects were observed on the OAW-42 cell line.

The RTCA curves for the exploratory antigen-blocking experimental points showed similar behavior to their respective points without antigen, for both resting and activated mRNA-CAR-T cells. This observation may be explained by the high production rate of CAR on the cell surface. However, because the physiological concentration of CA125 is measured in units that cannot be easily converted to weight, it is not possible to adequately interpolate these results to physiologic conditions.

RTCA is a convenient and precise method for evaluating cell viability, but it has a key limitation: it measures cell adhesion rather than immediate viability. To confirm functional antitumor activity using an orthogonal method, total metabolic activity of cancer cells cultured with CA125-specific CAR-T cells was evaluated using a CCK-8 assay. The CCK-8 assay was performed with activated and resting cells derived from two donors as effectors and OVCAR-3 and OAW-42 cells as targets (Supplementary Figure S11). Compared with pulse-control co-cultures, co-cultures containing CA125-specific CAR-T cells showed a reduced CCK-8 signal in the presence of OVCAR-3 cells, whereas no comparable reduction was observed with OAW-42 cells. The same reduction was observed in assays with plasmid-derived CAR-T cells. No signal reduction was observed in wells with OAW-42 cells. Although CCK-8 assays cannot distinguish effector from target signals, the marked decrease in signal may indicate cytotoxic activity in the CAR-T cells studied.

TNF-α levels were measured in wells after the CCK-8 cytotoxicity assay (Supplementary Figure S12). OAW-42 induced less TNF-α secretion than OVCAR-3 in both resting and activated lymphocytes, regardless of CAR expression. Similarly, activated T-cells secreted elevated levels of TNF-α regardless of stimulation or CAR expression. The immunogenicity of OVCAR-3 may partially explain the cytotoxic effect observed in activated pulse-control cells.

### 3.6. CAR mRNA-Electroporated T Cells Exhibit OVCAR-3-Dependent IFN-γ, IL-2, and Granzyme B Responses

The functional activity of activated and resting T cells following CAR mRNA electroporation was assessed by simultaneous enumeration of IFN-γ-, IL-2-, and granzyme B-secreting cells (Figure 7).

At 5 h, pulse-only controls displayed minimal or no OVCAR-3-dependent IFN-γ and IL-2 responses. Activated pulse-only cells produced comparable numbers of IFN-γ spots in response to OVCAR-3 target cells and OAW-42 control cells (79 and 76 SFU per 10^4^ T cells, respectively), whereas resting pulse-only cells exhibited only background-level responses (9 and 6 SFU per 10^4^ T cells, respectively).

A modest OVCAR-3-associated granzyme B response was detected in activated pulse-only cells, while granzyme B secretion by resting pulse-only cells remained negligible.

Electroporation with CAR mRNA markedly increased the numbers of IFN-γ-, IL-2-, and granzyme B-secreting cells in response to OVCAR-3 relative to OAW-42. At 5 h, activated CAR mRNA-electroporated T cells generated 327 IFN-γ SFU per 10^4^ T cells in response to OVCAR-3 compared with 68.5 SFU in response to OAW-42.

Resting CAR mRNA-electroporated cells also demonstrated a pronounced target cell-dependent IFN-γ response, with 180.5 SFU per 10^4^ T cells against OVCAR-3 and 18.5 SFU against OAW-42. Activated CAR mRNA-electroporated cells additionally exhibited robust OVCAR-3-associated granzyme B and IL-2 responses. Resting CAR mRNA-electroporated cells showed a substantial IFN-γ response but comparatively lower granzyme B and IL-2 responses at this early time point.

At 10 h, when only CAR mRNA-electroporated cells were evaluated, OVCAR-3-dependent responses remained evident in both activated and resting populations. Activated CAR mRNA-electroporated cells generated 465 IFN-γ SFU per 10^4^ T cells against OVCAR-3 compared with 106 SFU against OAW-42. Resting CAR mRNA-electroporated cells generated 205 and 25.5 IFN-γ SFU per 10^4^ T cells against OVCAR-3 and OAW-42, respectively.

The granzyme B response in activated CAR mRNA-electroporated cells was already high at 5 h and remained comparable at 10 h, consistent with rapid cytotoxic granule release. In resting CAR mRNA-electroporated cells, OVCAR-3-associated granzyme B and IL-2 responses increased between 5 and 10 h, indicating time-dependent acquisition or recruitment of these effector functions.

However, because activated and resting cells were evaluated at different E:T ratios (1:1 and 5:1, respectively), their absolute SFU values cannot be directly compared to determine the effect of prior T-cell activation.

Collectively, these findings show that CAR mRNA electroporation was associated with an OVCAR-3-dependent secretory and degranulation-related response in both activated and resting T cells.

## 4. Discussion

Previous studies have described anti-CA125/MUC16 CAR-T cells [12,38] and mRNA-based CAR-T manufacturing strategies [32,34,49]. This study evaluates the combination of a selected anti-CA125 CAR with mRNA electroporation and compares manufacturing conditions using activated and resting T-cells. The validity of “fast” and “ultra-fast” mRNA-CAR-T manufacturing timelines of 2 and 4 days, respectively, is demonstrated, whereas other protocols require an 8-day process [49]. The use of fresh cells or omission of overnight post-thaw recovery may further reduce manufacturing time to less than 24 h. CAR-T therapy for solid tumors remains in its early stages, and multiple strategies are being developed to address the associated challenges [28]. A central issue is balancing efficacy and safety. In certain anti-CD19 CAR-T settings, increased persistence correlates with more durable activity, although this relationship is influenced by CAR design, disease context, and host factors [51,52]. If a tumor-associated antigen is also present in healthy tissues, prolonged persistence increases the risk of damaging those tissues, which express the antigen at lower levels. To enhance safety and specificity, strategies such as inducible apoptosis [53], logical CARs activated only in the presence or absence of specific antigens [54], CARs with modified affinity [55], hypoxia-activated CARs [56], or protease-activated CARs [57] have been proposed. Transient T cell modification using mRNA or plasmid DNA is another approach. While local administration and transient CAR expression may limit the duration of target recognition, they do not eliminate the risk of acute on-target/off-tumor effects or cytokine-mediated toxicity; these safety parameters were not evaluated in this study [34,35]. mRNA is a promising option for generating transient CAR-T cells because it does not require stable genomic integration and enables rapid protein expression [32,33]. Additionally, mRNA delivery via lipid nanoparticles, both in vitro and in vivo [58], may render electroporation systems unnecessary. Manufacturing time is a critical factor in the design and administration of CAR-T therapy. Non-viral delivery technologies facilitate the production of “fast” CAR-T products by bypassing the ex vivo expansion phase [37], reducing manufacturing time to 3–4 days. The “ultra-fast” CAR-T process further reduces this timeline to 1 day by omitting the activation phase [36]. In this study, the functionality of “ultra-fast” CAR-T cells transfected with mRNA and manufactured from cryopreserved material within 2 days was demonstrated. However, the cytotoxicity of “ultra-fast” CAR-T cells derived from resting cells was lower than that of CAR-T cells derived from activated T-cells. Under the electroporation and culture conditions tested, activated T-cells exhibited greater in vitro activity than resting T-cells, although activation status and pulse program were not independently assessed. The treatment of solid tumors necessitates the development of new technical solutions for CAR-T therapy [33]. For instance, transgenic chemokine receptors can enhance the infiltration of tumors by adoptively transferred T-cells [28]. Additional modifications, such as knocking out checkpoint molecules on T lymphocytes [59] or delivering immunomodulatory cytokines to the tumor via oncolytic viruses [60], are required to overcome the immunosuppressive tumor microenvironment. CAR-T therapy without these modifications may be effective only within specific clinical niches. The intended application for the CAR-T cells developed in this study is post-operative therapy for ovarian cancer, where surgical removal of the largest tumors is followed by CAR-T-mediated elimination of single disseminated cells and small metastases that lack an established immunosuppressive microenvironment. Intraperitoneal administration has demonstrated greater efficacy for treating peritoneal carcinomatosis compared to intravenous administration, due to improved access of CAR-T cells to tumor cells [31]. A significant concern regarding mRNA-CAR-T cells is the narrow cytotoxicity window. CAR protein expressed from linear transgenic mRNA typically peaks within the first 24 h and declines rapidly, becoming undetectable after 3 to 5 days [61]. Nevertheless, short-lived mRNA-CAR-T cells have demonstrated efficacy in preclinical models, such as glioblastoma, when strategies like repeated infusions, locoregional delivery combined with surgical extraction, and simultaneous targeting of multiple antigens are employed [62]. Another concern is the potential for soluble antigen to act as a decoy, reducing the amount of available CAR on the cell surface [63]. However, in a preclinical in vivo model, soluble CA125 did not impair the efficacy of CA125-specific CAR-T therapy [12].Another important consideration is the potential on-target off-tumor activity of CA125-targeted CAR-T therapy. While it may theoretically occur because MUC16 is expressed on bronchial, endometrial, ovarian, and corneal epithelial cells [64] in healthy tissues, its expression is limited to the apical terminus of the cell [16,17]. In theory, this makes contact between the T cell and the exposed antigen less likely; in cancer, however, dysregulated cell polarity makes MUC16 more accessible to CAR-T cells [65]. In real clinical settings, no off-tumor effects of MUC16-targeted CAR-T have been reported [66]. Current evidence for real off-tumor activity of CA125-targeted CAR-T is scarce and warrants further study.At this stage, only in vitro research has been conducted, and whether transient mRNA expression of this CAR-T product is sufficient to confer efficacy remains unresolved without animal model data. An in vivo study assessing the efficiency of CA125-specific mRNA is currently ongoing. The precise epitope recognized by the CAR is unknown, precluding adequate epitope-blocking experiments. The model also lacks a genetically matched antigen-loss control, and the negative control cell line is not isogenic, which may have influenced the observed results. As an exploratory study, the CAR-T product has not been tested against patient-derived tissue samples, ascites, or healthy tissue samples. A direct comparison of mRNA-CAR-T cells with lentiviral CAR-T cells would have strengthened the findings. This study also highlights opportunities to facilitate mRNA CAR-T manufacturing and application. Our previous research demonstrated that mRNA-CAR-T cells can exert cytotoxic activity as early as 2 h after transfection [67]. Furthermore, the manufactured product can be cryopreserved 2 h post-transfection without significant loss of effector function. Collectively, these findings underscore the high potential of mRNA-based CAR-T technology.

## 5. Conclusions

This study provides preliminary in vitro evidence that human T cells can be electroporated with mRNA encoding an anti-CA125 CAR and can display activity against a CA125/MUC16-high ovarian cancer cell line. Under the tested conditions, activated cells showed greater activity than nonactivated cells, while nonactivated cells retained measurable activity after rapid manufacture. The mRNA and plasmid products differed in cytotoxicity in the present experimental system, but this comparison requires validation using matched constructs, normalized CAR expression, viability measurements, and independent donors. The potential safety advantage, persistence profile, and postoperative application of this approach remain to be established in additional preclinical models.

## Figures and Tables

**Figure 1:**
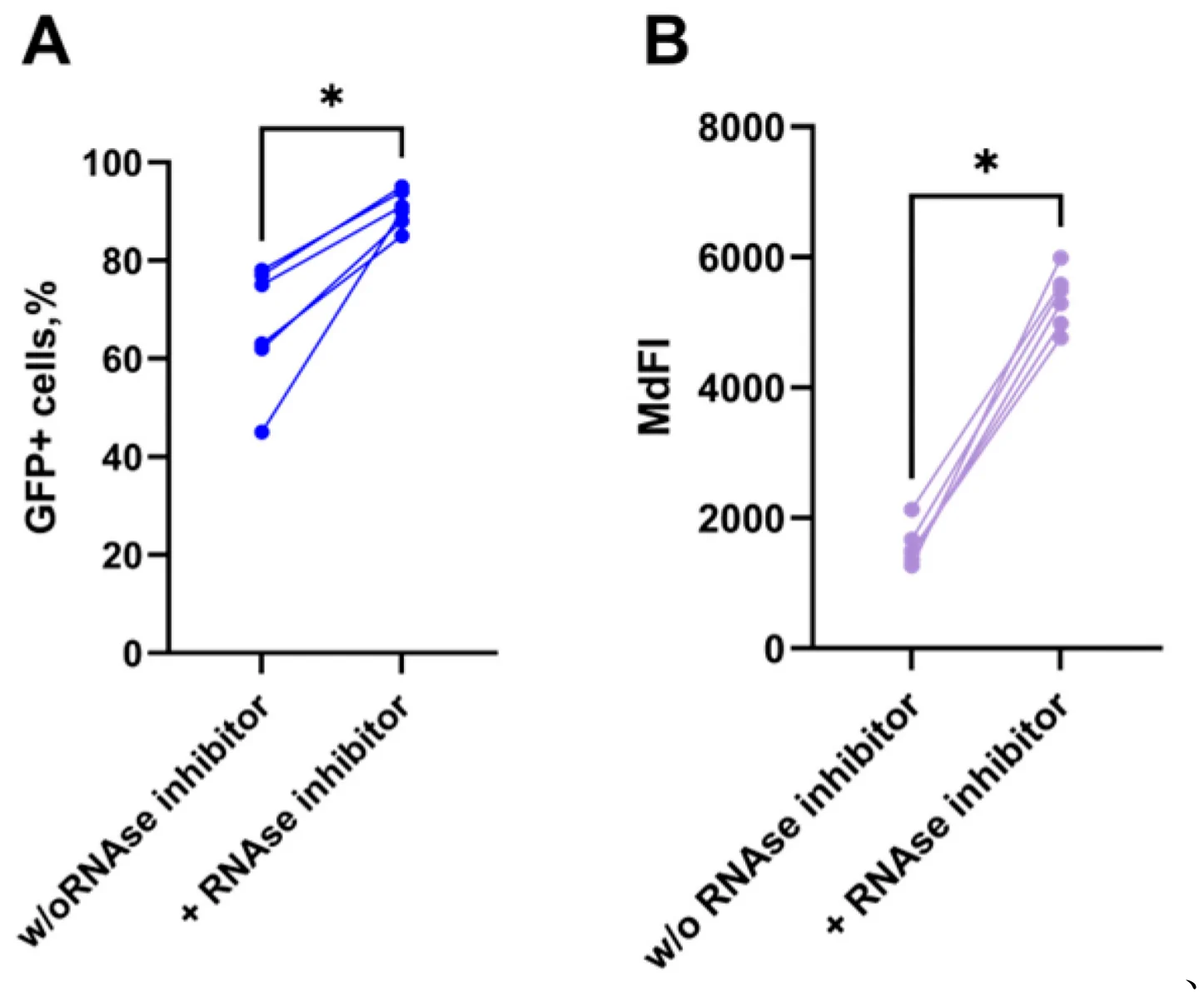
RNase inhibitors enhance transgene expression during mRNA transfection in PBMCs from healthy donors. (**A**) RNase inhibitors significantly increase the percentage of GFP+ cells (*, *p* = 0.031). (**B**) RNase inhibitors elevate MdFI by more than threefold (*, *p* = 0.031). Statistical significance was determined using the paired nonparametric Wilcoxon test. Data represent 6 independent experiments on paired samples from 6 donors.

**Figure 2:**
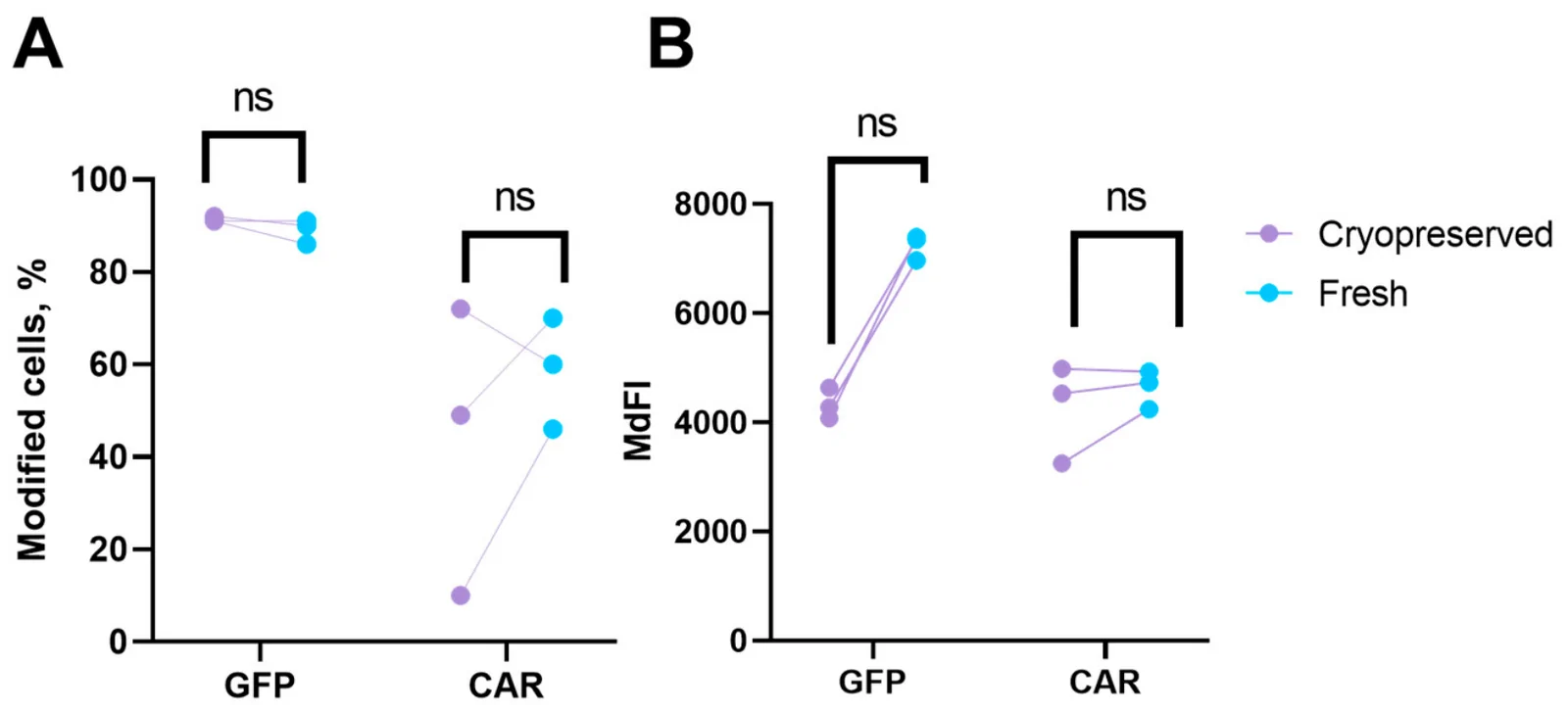
Effect of cryopreservation on mRNA transfection efficiency and transgene expression. (**A**) Cryopreservation of the starting material does not significantly affect the percentage of modified cells (ns, *p* = 0.5). (**B**) Analysis of three paired donors reveals no statistically significant difference between fresh and cryopreserved samples (*p* = 0.5). Statistical significance was assessed using the Wilcoxon signed-rank test; ns indicates *p* > 0.05. Data represent 3 independent experiments on paired samples from 3 donors.

**Figure 3:**
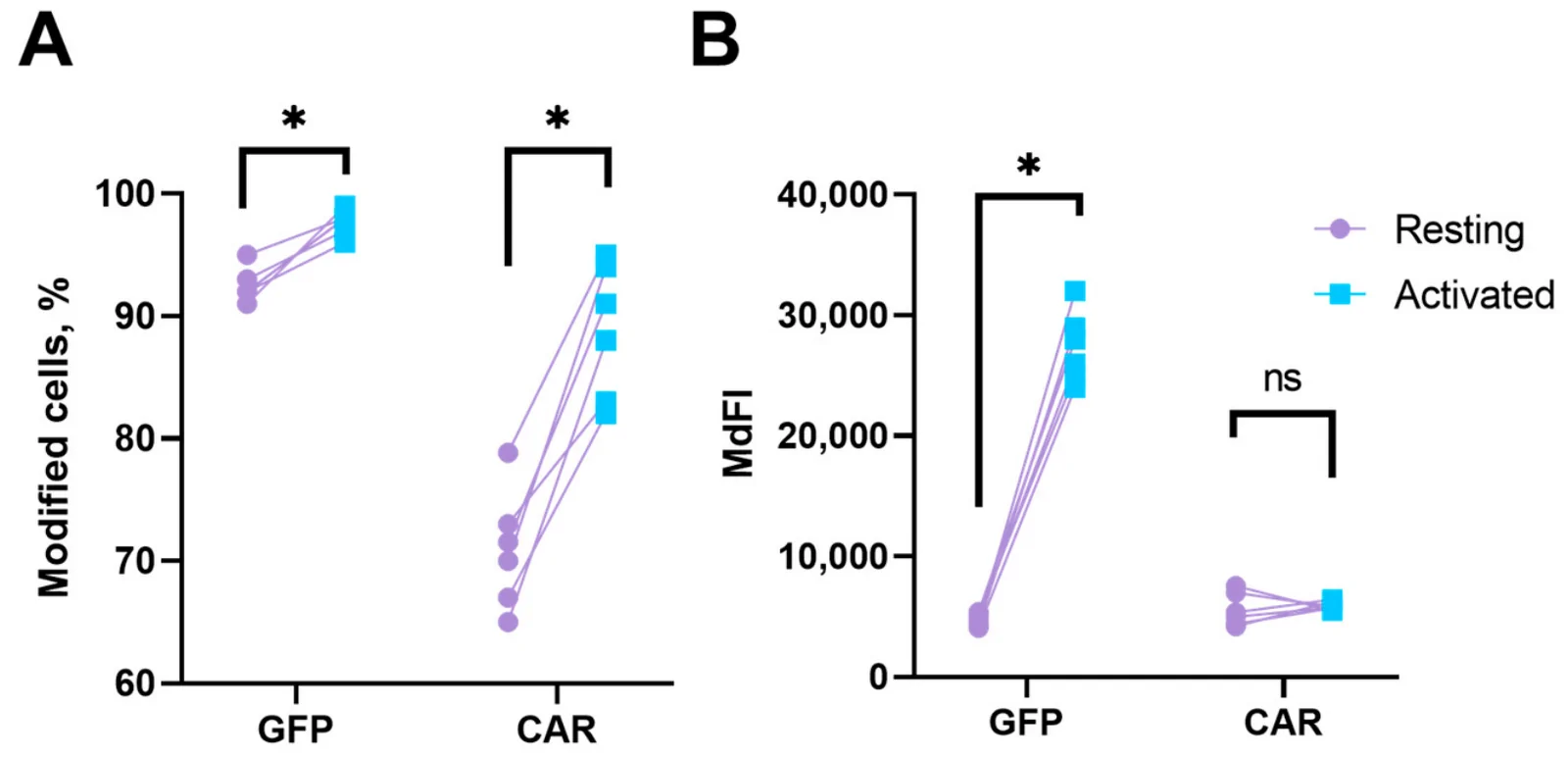
T-lymphocyte activation enhances mRNA transfection efficiency. (**A**) Activation increases modification efficiency for both GFP (*p* = 0.031) and CAR (*p* = 0.031). (**B**) Activation significantly enhances GFP expression (*p* = 0.031), but not CAR expression (*p* = 0.843). Statistical significance was determined using the Wilcoxon signed-rank test; ns indicates *p* > 0.05, * indicates *p* = 0.031. Data represent 6 independent paired experiments on cells from 6 donors.

**Figure 4:**
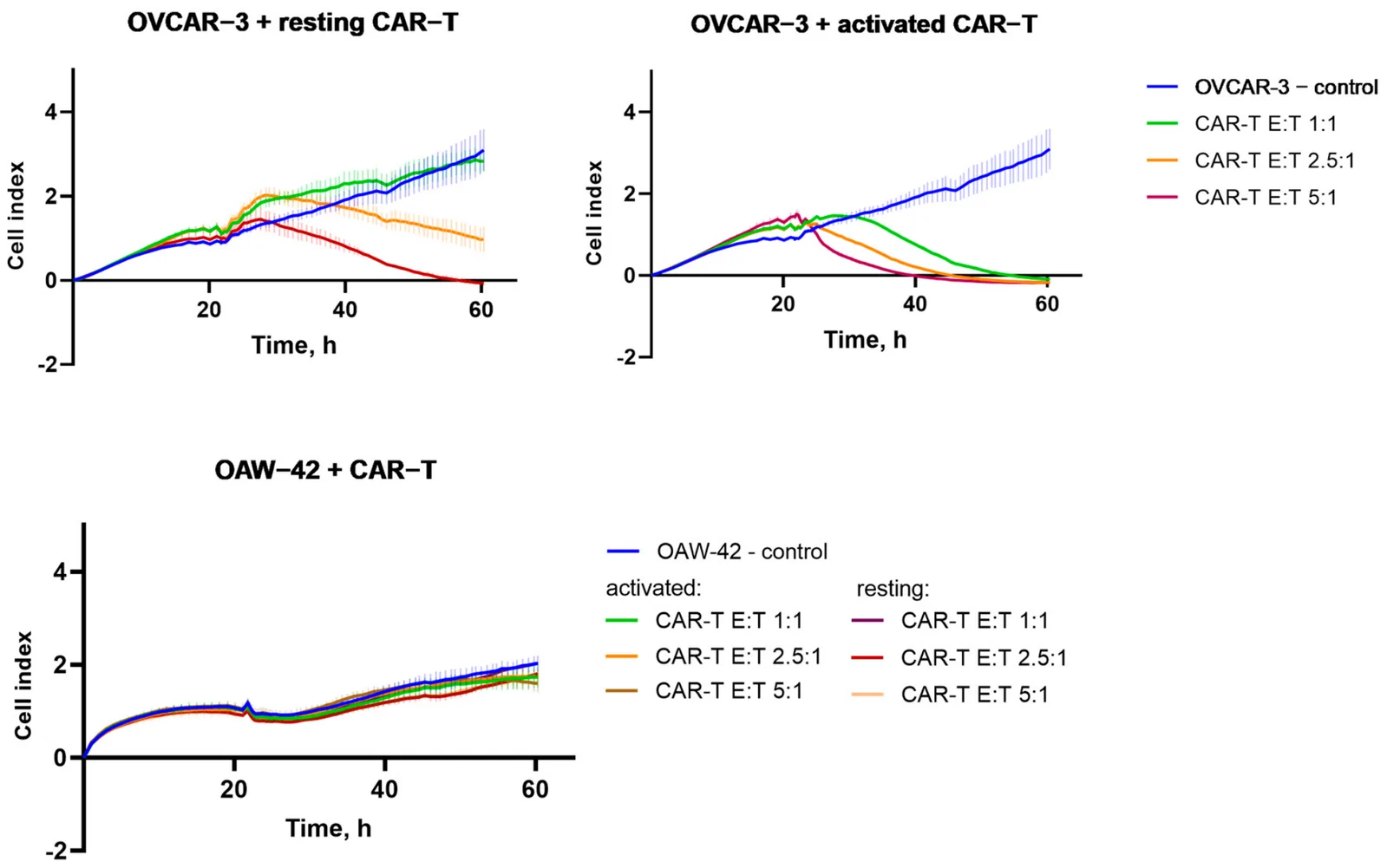
“Fast” and “ultra-fast” CAR-T cells exhibit cytotoxicity against the ovarian cancer cell line OVCAR3, characterized by high CA125 expression. (**top**) Cytotoxic effects are observed when either resting (“ultra-fast” CAR-T) or activated (“fast” CAR-T) cells are added to OVCAR-3. (**bottom**) No cytotoxicity is detected when CAR-T cells are added to the surface MUC16-negative cell line OAW-42. Data represent 1 donor. Graphs show the mean of two technical replicates ± SEM.

**Figure 5:**
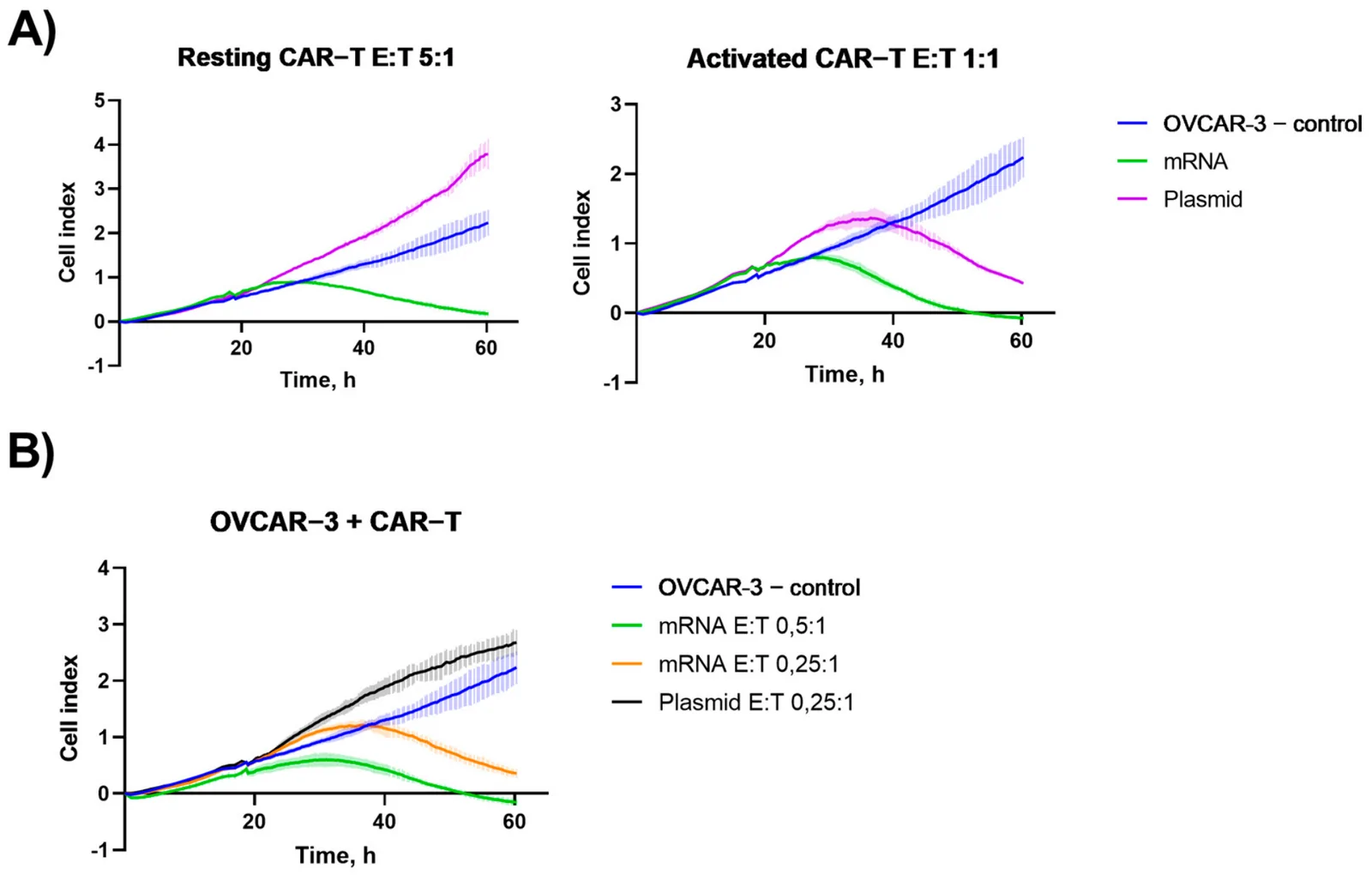
CA125-specific mRNA-CAR-T cells outperform the cytotoxic activity of plasmid-transfected CAR-T cells under the tested conditions. (**A**) Resting CAR-T cells are effective only at the E:T ratio 5:1, while activated CAR-T cells are effective even at a 1:1 E:T ratio. (**B**) Activated CAR-T cells are effective at the E:T ratio as low as 1:4. Data: 1 donor. Graphs represent the mean between two technical replicates ± SEM.

**Figure 6:**
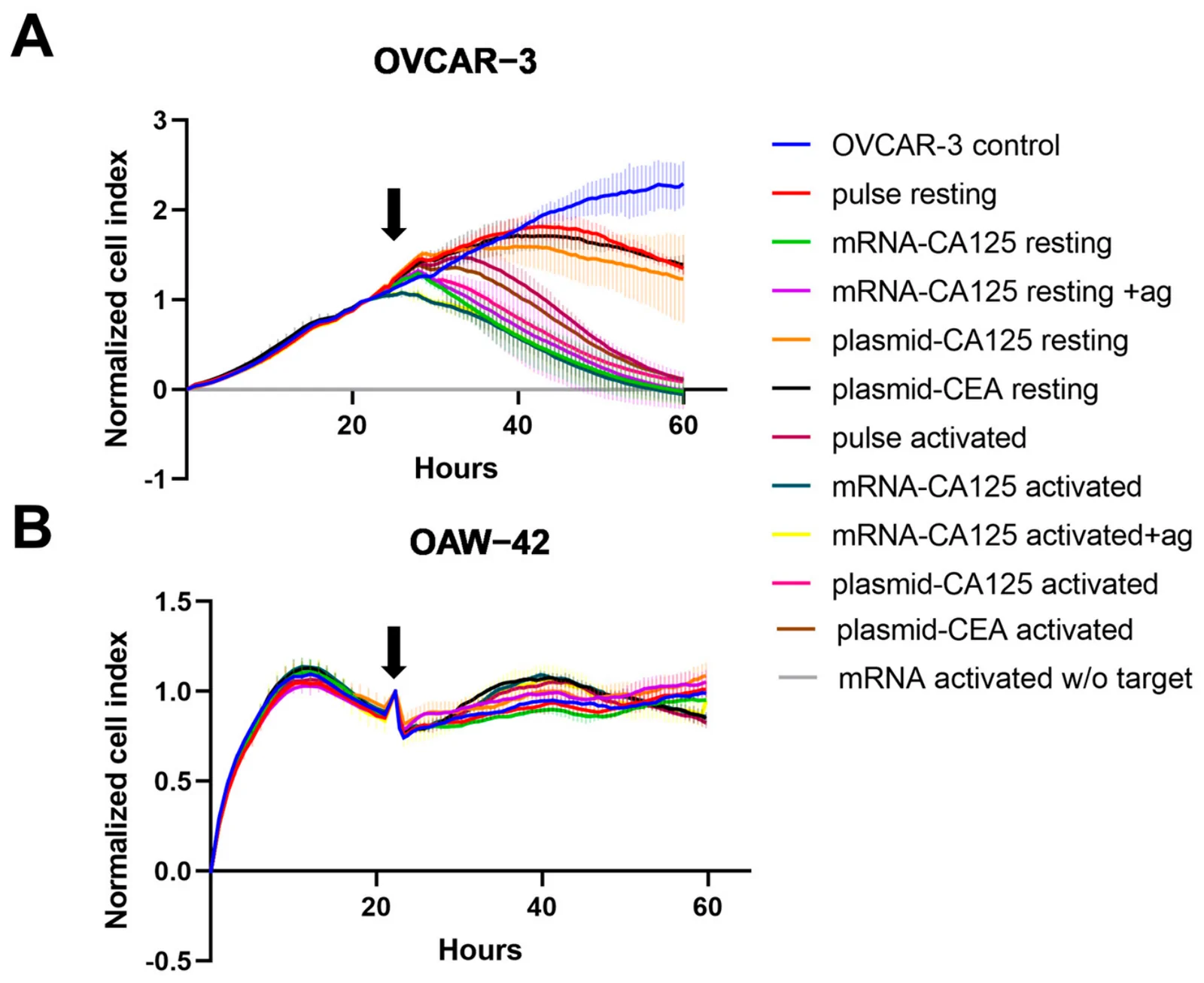
CA125-specific CAR-T cells exhibit time-dependent cytotoxic activity across three different donors. (**A**) CAR-T cells demonstrate cytotoxicity against the OVCAR-3 cell line. (**B**) No cytotoxic effect was observed on the OAW-42 cell line. Plasmid and mRNA CAR-T cells were generated from both activated and resting T-cells. The E:T ratio was 5:1 for resting T-cells and 1:1 for activated T-cells. Pulse-control and irrelevant (CEA-specific) CAR controls were included. An additional experiment with blocking by soluble CA125 antigen was conducted (+ag). Two-pointed arrows indicate statistical differences between pulse-control and mRNA-transfected CAR. A single-sided arrow marks the time point of effector addition. Data represent 3 independent experiments performed on cells from 3 donors; each experimental point for any single donor was performed in two technical replicates. Graphs show the mean of three independent experiments for mRNA and two independent experiments for plasmid ± SEM.

**Figure 7:**
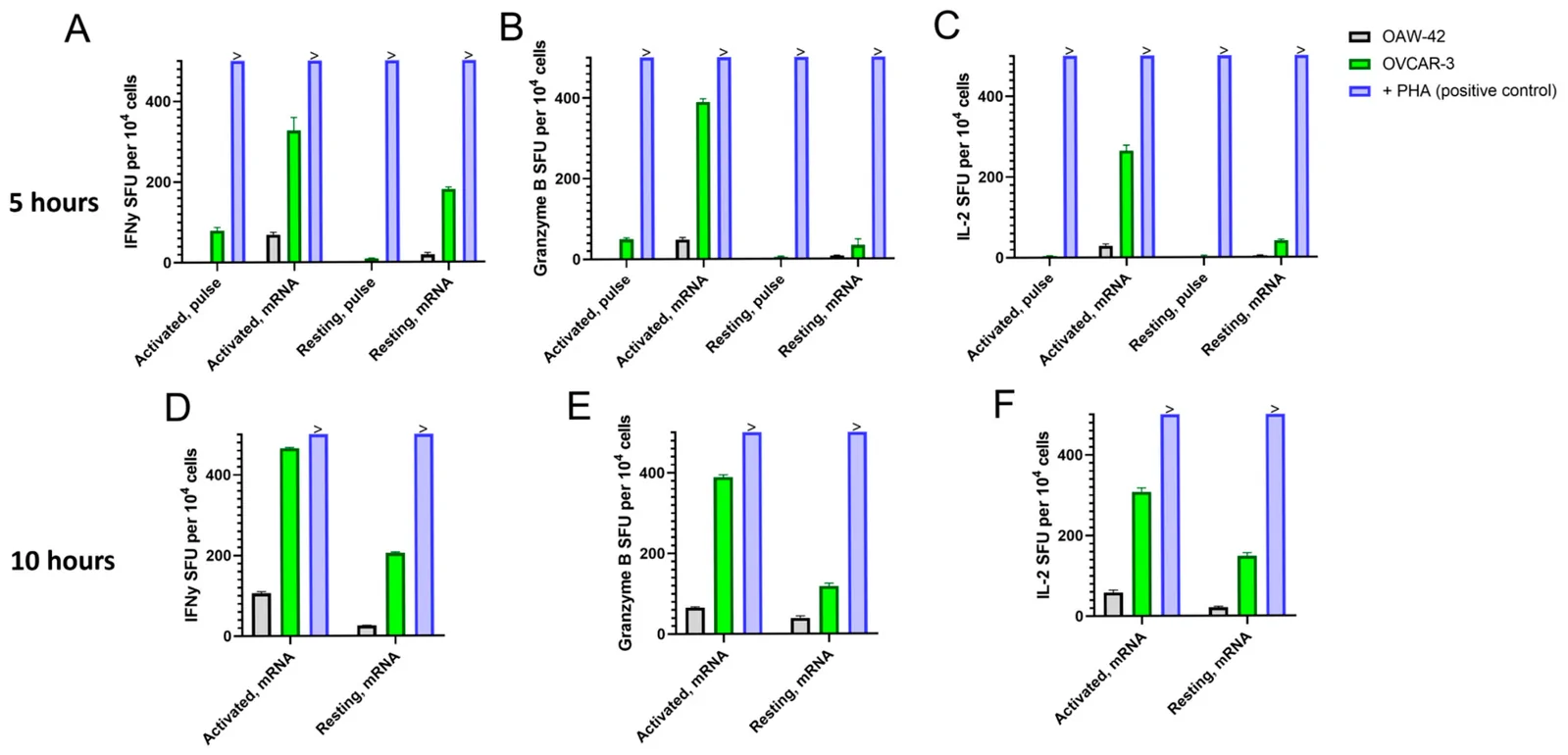
Target cell-dependent IFN-γ, IL-2, and granzyme B responses of activated and resting CAR mRNA-electroporated T cells. Activated or resting T cells were electroporated without mRNA (pulse) or with CAR-encoding mRNA (mRNA) and subsequently co-cultured with OVCAR-3 target cells (green bars) or OAW-42 control cells (gray bars). Activated T cells were co-cultured with tumor cells at an E:T ratio of 1:1, whereas resting T cells were tested at an E:T ratio of 5:1. PHA-stimulated cells served as positive controls (violet bars). The numbers of IFN-γ-, granzyme B-, and IL-2-secreting cells were determined after 5 h (**A**–**C**) or 10 h (**D**–**F**) using a three-color FluoroSpot assay. Bars represent the mean ± SD of technical duplicate wells. The “>” symbol above the PHA bars indicates responses exceeding 500 SFU per 10^4^ effector T cells.

## Data Availability

The original contributions presented in this study are included in the article/Supplementary Materials. Further inquiries can be directed to the corresponding author.

## Supplementary Materials

The following supporting information can be downloaded at https://www.mdpi.com/article/10.3390/biomedicines14102147/s1, Figure S1: Purity of the CD3:CD8+ fraction after magnetic separation reaches 97%. Representative examples for donors D17 and D18 are shown; Figure S2: Scheme of CAR construct cloned in a plasmid. mRNA vector contained the identical CAR sequence; Figure S3: Pipeline of CAR-T manufacturing from activated and resting T-cells. PBMCs are isolated from blood concentrate using Ficoll gradient centrifugation and then cryopreserved. For the activation pipeline (**A**), cryopreserved cells are thawed, and the next day CD3+ cells are magnetically separated and stimulated with CD3/CD28 beads and IL-2. After 2 days of activation, cells are electroporated with mRNA or plasmid DNA encoding CAR. The next day after electroporation, effector qualities of CAR-T cells are measured using real-time cell analysis (RTCA) and CCK-8 assays. In the pipeline without activation (**B**) and (**C**), cells are electroporated the same day after magnetic separation. The use of fresh material (**C**) allows the CAR-T manufacturing within the 24 h. For the functional comparison of the CAR-T product, cells from the same donors were used. Electroporation and functional assays were conducted in parallel for resting and activated cells; Figure S4: Gating strategy for the CAR modification efficiency assessment using flow cytometry. (A) Lymphocytes are gated by FSC/SSC. (B) The DAPI stain distinguishes live cells. (C) Electroporation without plasmid DNA was used as a negative control. (D) A representative example of a CAR-T cellsnstained with the CA125 antigen; Figure S5: Flow cytometry analysis of MUC16 expression on OVCAR-3 and OAW-42 cell lines; Figure S6: Phenotypic characterization of activated (A) and resting (B) T-cells, electroporated with either CAR-encoding mRNA, plasmid, or pulse control. Electroporation elevated CD69 expression in resting cells. In activated cells, CD69, CD25, and PD1 levels increased after CD3/28 and IL-2 stimulation and were unaffected by electroporation. Measurements were taken on Day 1, after CD3+ magnetic separation; Day 2 for resting cells (the day after electroporation); Day 3 for activated cells (immediately before electroporation); and Day 4 for activated cells (the day after electroporation). Data: 2 donors. Each point represents one donor; the horizontal bars represent the mean between two donors; Figure S7: Expression dynamics of CAR observed for 7 days. PBMCs from one healthy donor were used. Transgene expression declines more rapidly in activated cells; plasmid DNA provides longer transgene expression than mRNA. Data: 1 donor; Figure S8: Dose-dependent dynamics of mRNA and plasmid modification. Resting and activated PBMCs from one donor were transfected with CAR-encoding mRNA and plasmid at doses of 1, 2.5, 5, 10, and 20 µg; Figure S9: CA125-specific CAR-T cells demonstrate time-dependent cytotoxic activity across 3 donors. Individual RTCA curves are shown separately for 3 donors. Plasmid and mRNA CAR-T cells were manufactured from activated and resting T cells. Graphs represent the mean between two technical replicates ± SEM. Pulse-control and irrelevant (CEA-specific) CAR controls were included. Different experiment numbers for plasmid and mRNA reflect the limited capacity of the xCELLigence device; to maintain the same experimental conditions, we omitted some experimental points. Additionally, we performed an experiment with blocking by soluble CA125 antigen (+ag). A, B, C—RTCA curves for lymphocytes originating from D17, D18, and D19, respectively, cultured with the OVCAR-3 cell line. D, E—RTCA curves for lymphocytes originating from D17 and D18, respectively, cultured with the OAW-42 cell line; Figure S10: AUC analysis of RTCA curves of 3 donors. Plasmid and mRNA CAR-T cells were manufactured from activated and resting T cells. Each point represents the AUC value for one donor normalized to the AUC of the control wells (OVCAR-3 only); error bars represent the SEM. Resting mRNA CAR-T cells show over a 50% reduction in AUC compared with pulse-control, confirming CAR efficiency. Activated cells show reduced AUC due to increased non-specific cytotoxicity; Figure S11: CCK-8 assay shows cytotoxicity of CA125-specific CAR-T cells against OVCAR-3, but not OAW-42. The fluorescence signal is normalized to the control wells, which contain only cancer cells. Each point represents the mean of two independent experiments; we studied pulsed control, mRNA-derived, and plasmid-derived CAR-T cells manufactured from activated or resting T cells. CAR-expressing cells showed reduced signal compared with pulse control when cocultured with OVCAR-3 (A), but not with OAW-42 (B). Data: 2 donors. Error bars represent the SEM; Figure S12: TNF-α secretion of T-lymphocytes stimulated by OVCAR-3 cells (A) and OAW-42 (B). OVCAR-3 cells induce greater levels of TNF-α compared to OAW-42. Activated lymphocytes produce elevated amounts of TNF-α regardless of CAR expression. Data: 2 independent series of experiments performed on cells from 2 donors. The plotted values are the mean between two technical replicates. Error bars are omitted for graph presentation convenience. Table S1: Donor characteristics and relevance to the respective assays. Samples were obtained from 20 donors (male and female) aged 25–50 years, with donor relevance indicated for each assay.

## Abbreviations

The following abbreviations are used in this manuscript:

ANOVA	analysis of variance
AUC	area under the curve
BSA	bovine serum albumin
CA125	cancer antigen 125
CAR	chimeric antigen receptor
CCK-8	Cell Counting Kit-8
CD	cluster of differentiation
CEA	carcinoembryonic antigen
DAPI	4′,6-diamidino-2-phenylindole
DMEM	Dulbecco’s modified Eagle medium
DMSO	dimethyl sulfoxide
DNA	deoxyribonucleic acid
E:T	effector-to-target ratio
EDTA	ethylenediaminetetraacetic acid
ELISA	enzyme-linked immunosorbent assay
FBS	fetal bovine serum
FITC	fluorescein isothiocyanate
FSC	forward scatter
GFP	green fluorescent protein
HBV	hepatitis B virus
HER2	human epidermal growth factor receptor-2
HIV	human immunodeficiency virus
IFN-γ	interferon gamma
IL-2	interleukin-2
MdFI	median fluorescence intensity
mRNA	messenger RNA
MUC16	mucin 16
PBMC	peripheral blood mononuclear cells
PBS	phosphate-buffered saline
PCR	polymerase chain reaction
PD-1	programmed cell death protein 1
PE	phycoerythrin
PerCP-Cy5.5	peridinin-chlorophyll-protein complex–cyanine 5.5
PHA	phytohemagglutinin
RNase	ribonuclease
RNA	ribonucleic acid
RPMI 1640	Roswell Park Memorial Institute 1640 medium
RTCA	real-time cell analysis
SD	standard deviation
SFU	spot-forming units
SSC	side scatter
STR	short tandem repeat
TCR	T-cell receptor
TNF-α	tumor necrosis factor alpha
