# Supplementary material for: Fast and Ultra-Fast mRNA-Engineered Anti-CA125/MUC16 CAR-T-Cells: An In Vitro Study
Source: Biomedicines. 2026 Sep 23;14(10):2147. doi: 10.3390/biomedicines14102147 (PMC13701628; doi:10.3390/biomedicines14102147)
Supplement: Supplementary file 1 [file biomedicines-14-02147-s001.zip › Supplementary data.pdf]

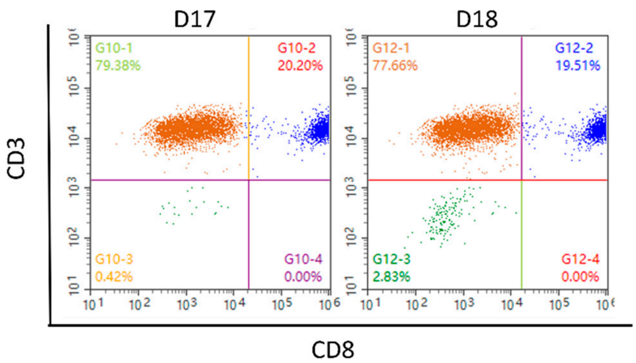

Supplementary Figure S1. Purity of the CD3+fraction after magnetic separation reaches 97%. Representative examples for donors D17 and D18 are shown.

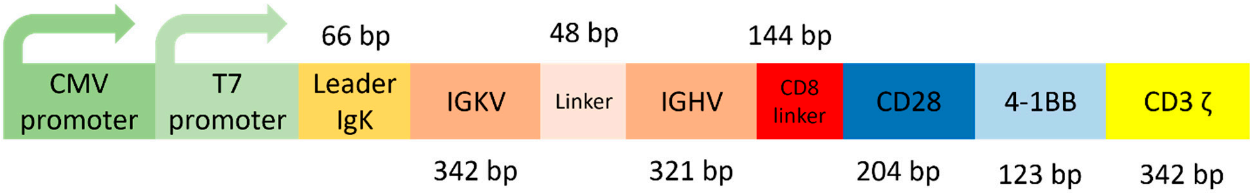

Supplementary Figure S2. Scheme of CAR construct cloned in a plasmid. mRNA vector contained the identical CAR sequence.

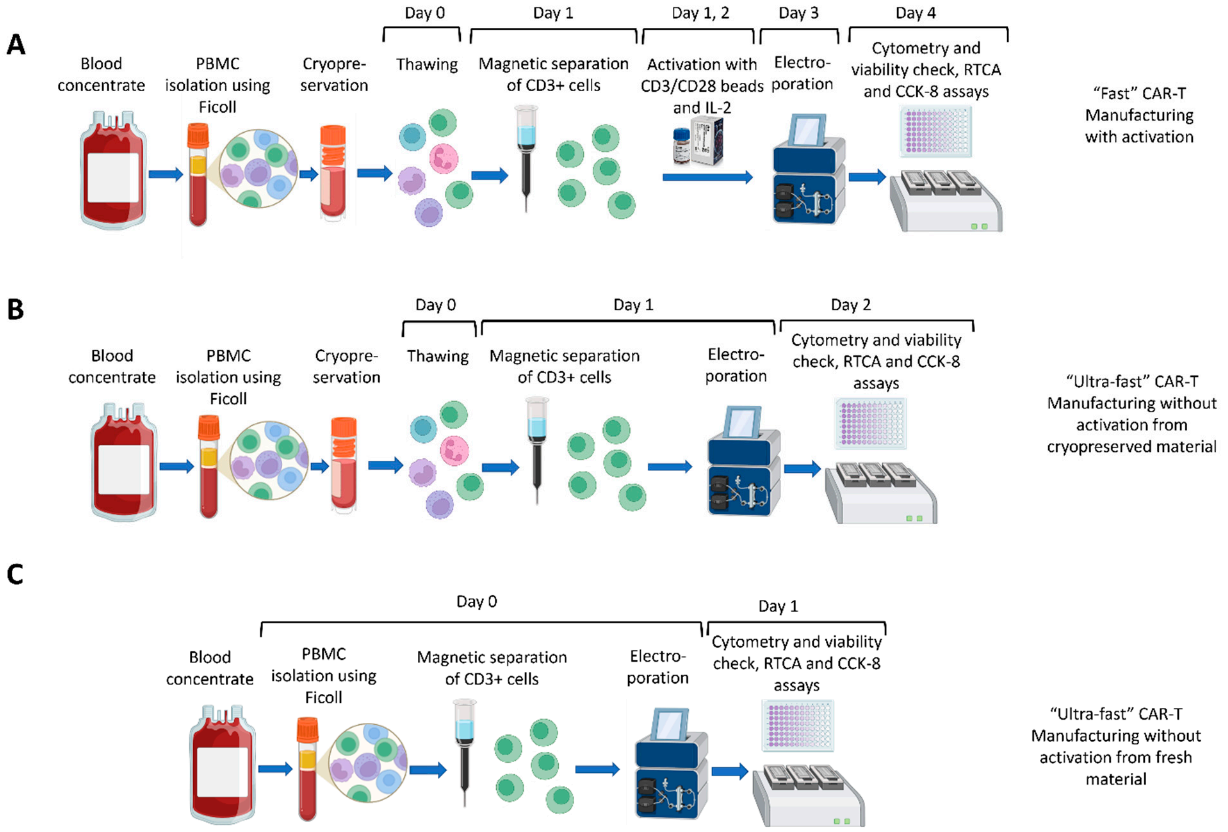

Supplementary Figure S3. Pipeline of CAR-T manufacturing from activated and resting T-cells. PBMCs are isolated from blood concentrate using Ficoll gradient centrifugation and then cryopreserved. For the activation pipeline **(A)**, cryopreserved cells are thawed, and the next day CD3+ cells are magnetically separated and stimulated with CD3/CD28 beads and IL-2. After 2 days of activation, cells are electroporated with mRNA or plasmid DNA encoding CAR. The next day after electroporation, effector

qualities of CAR-T cells are measured using real-time cell analysis (RTCA) and CCK-8 assays. In the pipeline without activation **(B)** and **(C)**, cells are electroporated the same day after magnetic separation. The use of fresh material **(C)** allows the CAR-T manufacturing within the 24 h. For the functional comparison of the CAR-T product, cells from the same donors were used. Electroporation and functional assays were conducted in parallel for resting and activated cells.

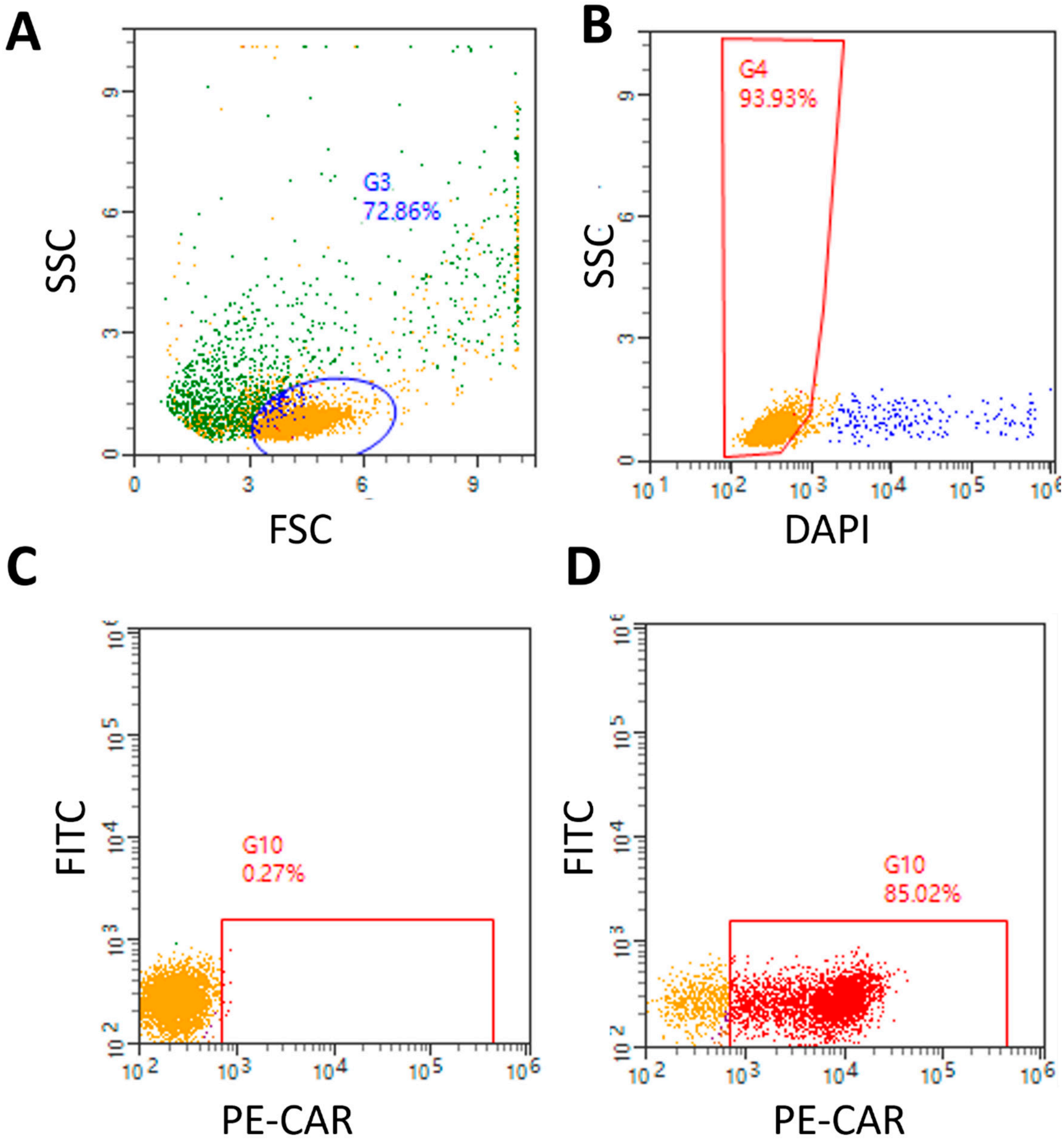

Supplementary Figure S4. Gating strategy for the CAR modification efficiency assessment using flow cytometry. A – Lymphocytes are gated by FSC/SSC. B – The DAPI stain distinguishes live cells. C – Electroporation without plasmid DNA was used as a negative control. D – A representative example of a CAR-T cellsnstained with the CA125 antigen.

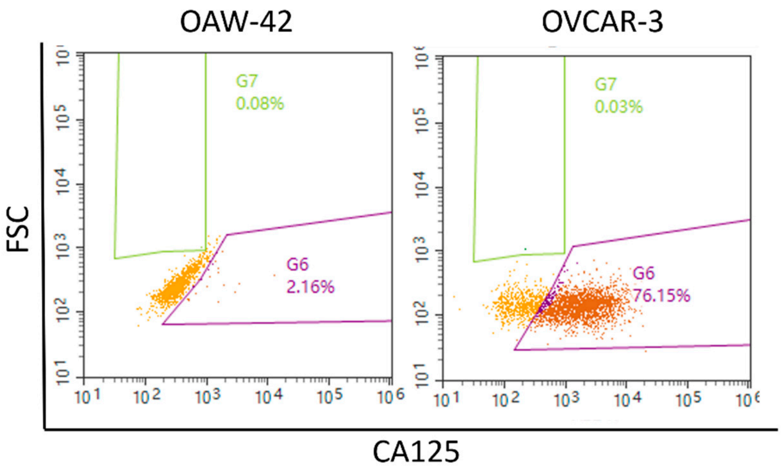

Supplementary Figure S5. Flow cytometry analysis of MUC16 expression on OVCAR-3 and OAW-42 cell lines.

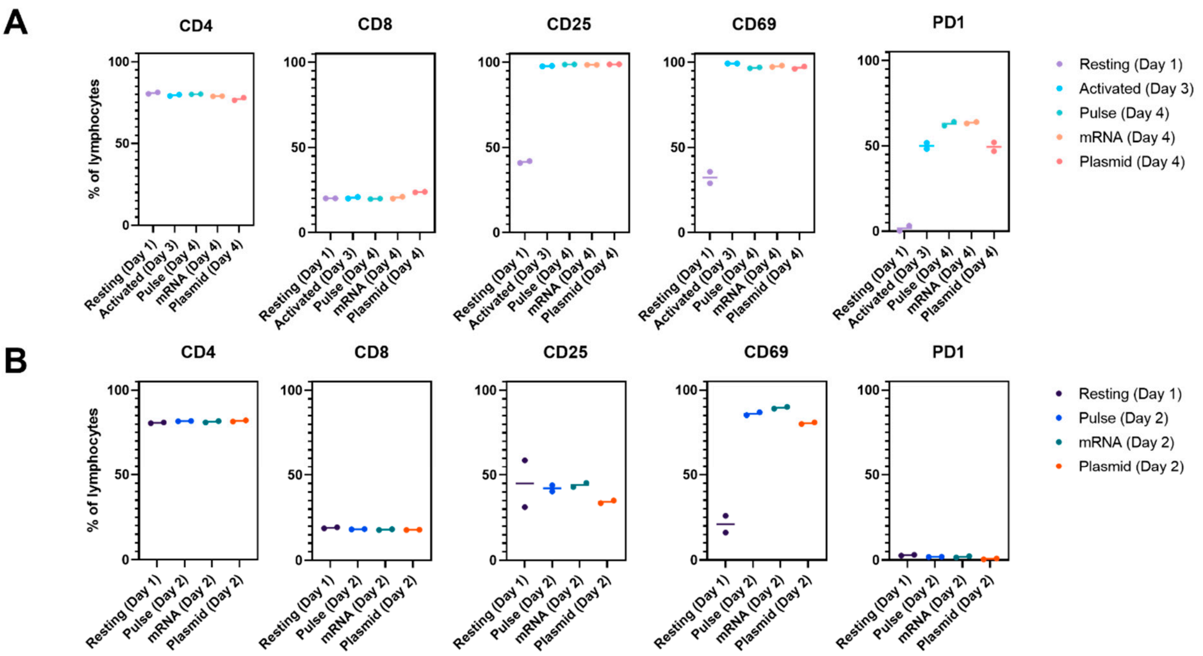

Supplementary Figure S6. Phenotypic characterization of activated (A) and resting (B) T-cells, electroporated with either CAR-encoding mRNA, plasmid, or pulse control. Electroporation elevated CD69 expression in resting cells. In activated cells, CD69, CD25, and PD1 levels increased after CD3/28 and IL-2 stimulation and were unaffected by electroporation. Measurements were taken on Day 1, after CD3+ magnetic separation; Day 2 for resting cells (the day after electroporation); Day 3 for activated cells (immediately before electroporation); and Day 4 for activated cells (the day after electroporation). Data: 2 donors. Each point represents one donor; the horizontal bars represent the mean between two donors.

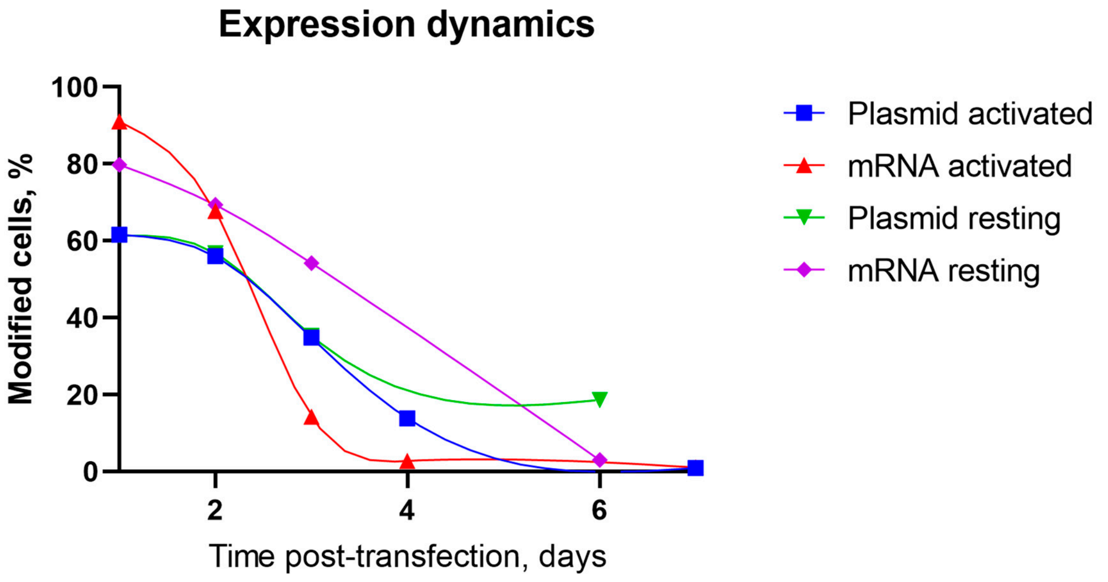

Supplementary Figure S7. Expression dynamics of CAR observed for 7 days. PBMCs from one healthy donor were used. Transgene expression declines more rapidly in activated cells; plasmid DNA provides longer transgene expression than mRNA. Data: 1 donor

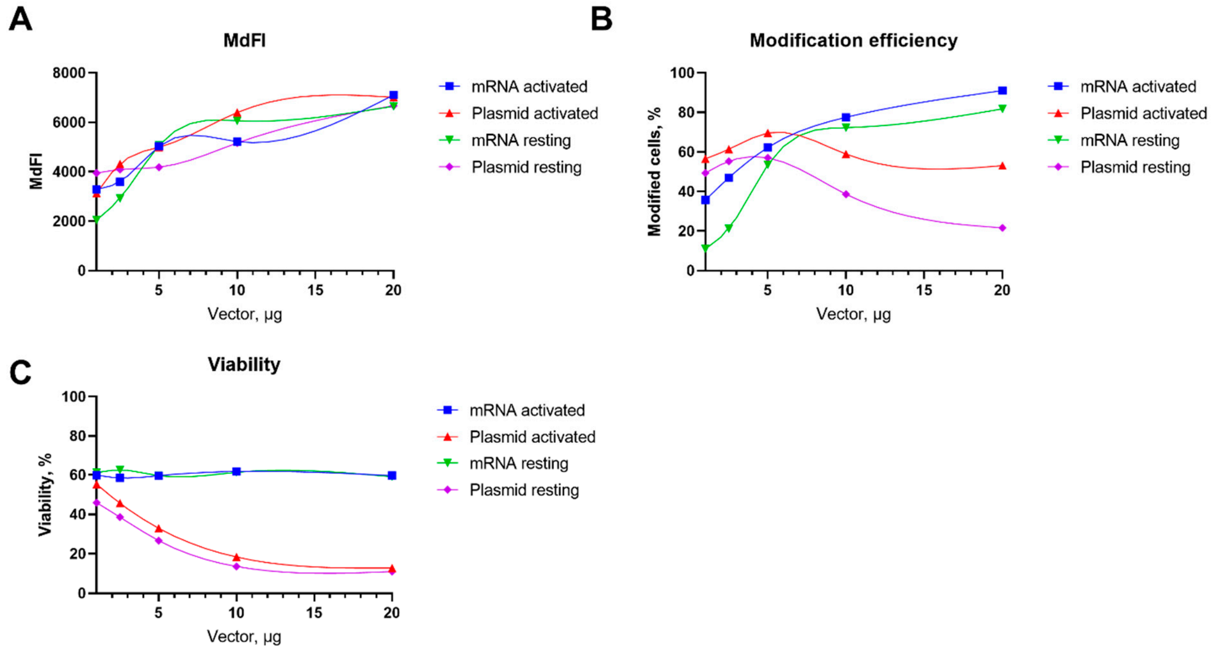

Supplementary Figure S8. Dose-dependent dynamics of mRNA and plasmid modification. Resting and activated PBMCs from one donor were transfected with CAR-encoding mRNA and plasmid at doses of 1, 2.5, 5, 10, and 20 μg.

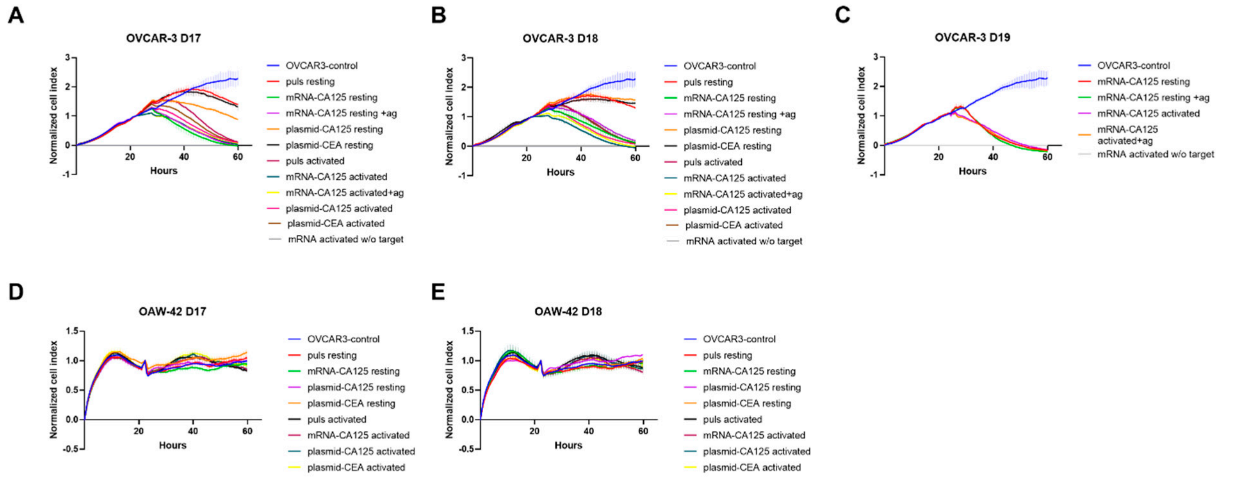

Supplementary Figure S9. CA125-specific CAR-T cells demonstrate time-dependent cytotoxic activity across 3 donors. Individual RTCA curves are shown separately for 3 donors. Plasmid and mRNA CAR-T cells were manufactured from activated and resting T cells. Graphs represent the mean between two technical replicates ± SEM. Pulse-control and irrelevant (CEA-specific) CAR controls were included. Different experiment numbers for plasmid and mRNA reflect the limited capacity of the xCELLigence device; to maintain the same experimental conditions, we omitted some experimental points. Additionally, we performed an experiment with blocking by soluble CA125 antigen (+ag). A, B, C – RTCA curves for lymphocytes originating from D17, D18, and D19, respectively, cultured with the OVCAR-3 cell line. D, E - RTCA curves for lymphocytes originating from D17 and D18, respectively, cultured with the OAW-42 cell line.

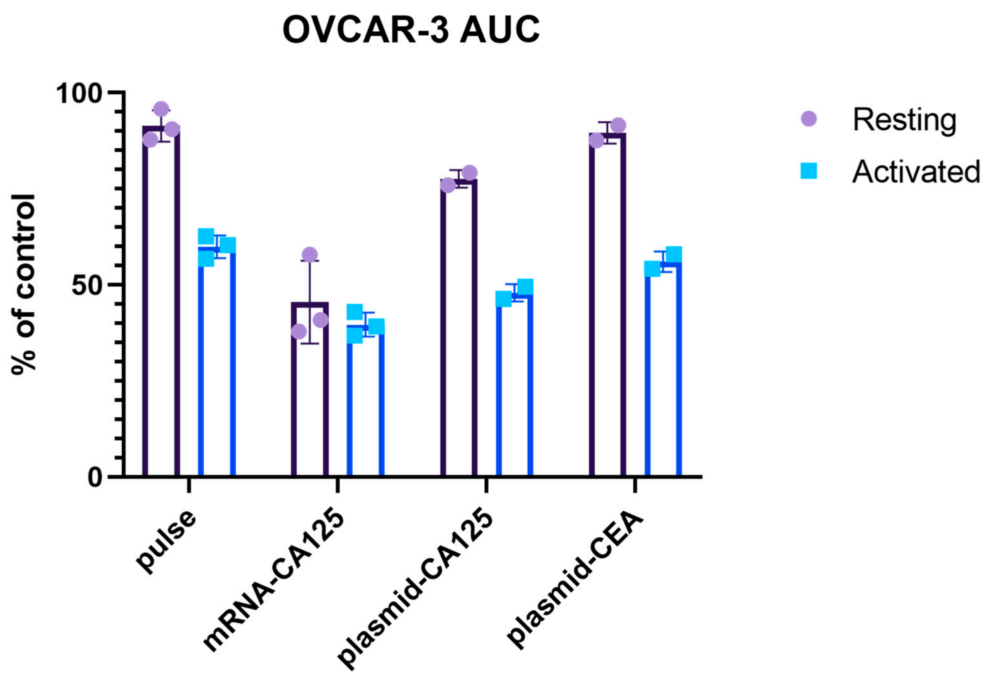

Supplementary Figure S10. AUC analysis of RTCA curves of 3 donors. Plasmid and mRNA CAR-T cells were manufactured from activated and resting T cells. Each point represents the AUC value for one donor normalized to the AUC of the control wells (OVCAR-3 only); error bars represent the SEM. Resting mRNA CAR-T cells show over a 50% reduction in AUC compared with pulse-control, confirming CAR efficiency. Activated cells show reduced AUC due to increased non-specific cytotoxicity.

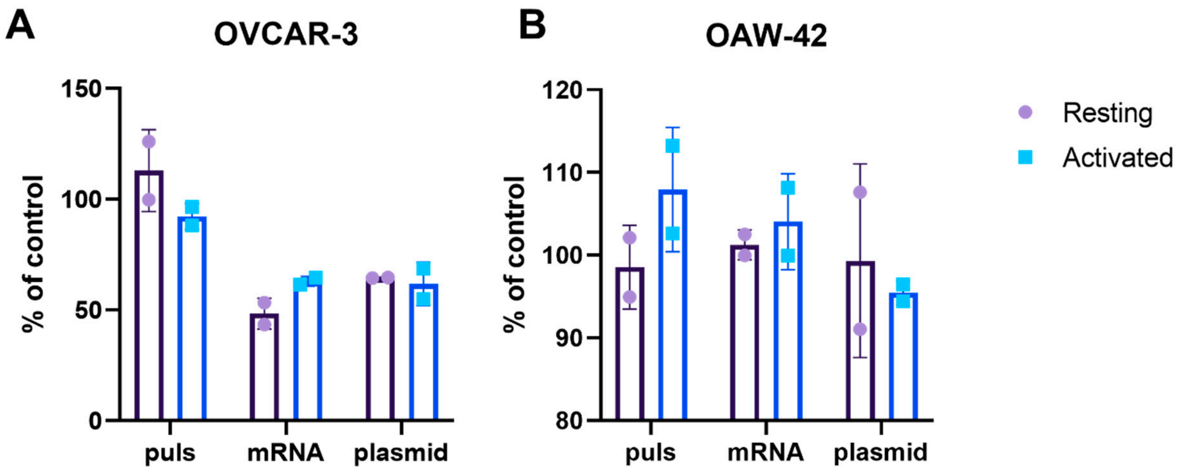

Supplementary Figure S11. CCK-8 assay shows cytotoxicity of CA125-specific CAR-T cells against OVCAR-3, but not OAW-42. The fluorescence signal is normalized to the control wells, which contain only cancer cells. Each point represents the mean of two independent experiments; we studied pulsed control, mRNA-derived, and plasmid-derived CAR-T cells manufactured from activated or resting T cells. CAR-expressing cells showed reduced signal compared with pulse control when cocultured with OVCAR-3 (A), but not with OAW-42 (B). Data: 2 donors. Error bars represent the SEM.

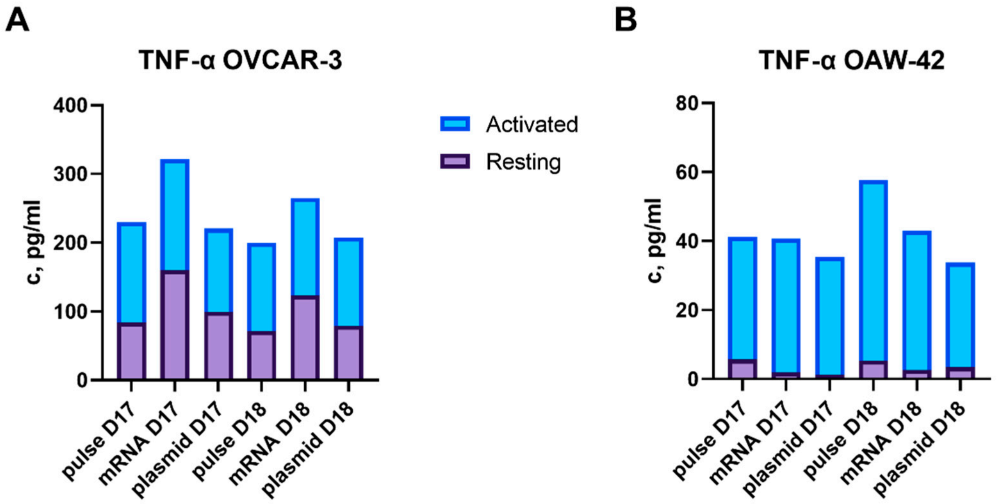

Supplementary Figure S12. TNF-α secretion of T-lymphocytes stimulated by OVCAR-3 cells (A) and OAW-42 (B). OVCAR-3 cells induce greater levels of TNF-α compared to OAW-42. Activated lymphocytes produce elevated amounts of TNF-α regardless of CAR expression. Data: 2 independent series of experiments performed on cells from 2 donors. The plotted values are the mean between two technical replicates. Error bars are omitted for graph presentation convenience.
